# Intracellular Synthesis of Indoles Enabled by Visible-Light Photocatalysis

**DOI:** 10.1021/jacs.3c13647

**Published:** 2024-01-26

**Authors:** Cinzia D’Avino, Sara Gutiérrez, Max J. Feldhaus, María Tomás-Gamasa, José Luis Mascareñas

**Affiliations:** Centro Singular de Investigación en Química Biolóxica e Materiais Moleculares (CIQUS), and Departamento de Química Orgánica, Universidade de Santiago de Compostela, 15705 Santiago de Compostela, Spain

## Abstract

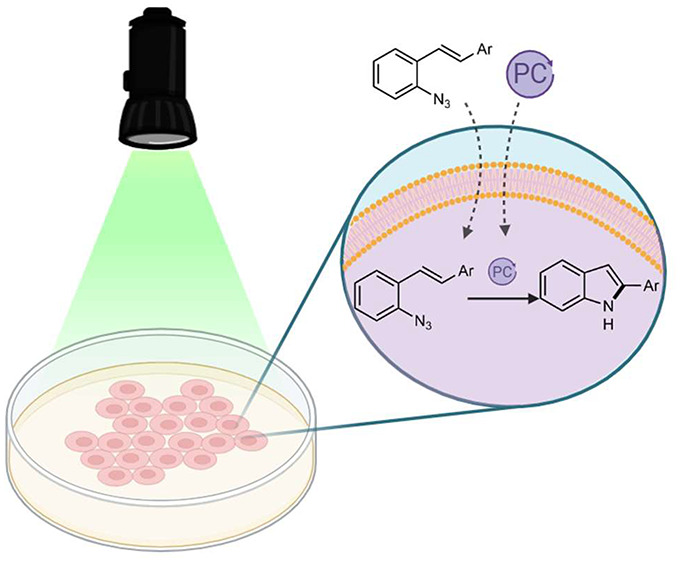

Performing abiotic synthetic transformations in live cell environments represents a new, promising approach to interrogate and manipulate biology and to uncover new types of biomedical tools. We now found that photocatalytic bond-forming reactions can be added to the toolbox of bioorthogonal synthetic chemistry. Specifically, we demonstrate that exogenous styryl aryl azides can be converted into indoles inside living mammalian cells under photocatalytic conditions.

The advent of photocatalysis has sparked a major revolution in synthetic organic chemistry.^1^ The rich reactivity patterns unlocked by photocatalysts generally arise from their ability to engage in electron-transfer (ET) or energy-transfer (EnT) processes after light irradiation (Figure 1A).^2^ At first sight, this type of reactivity might seem incompatible with aqueous media, and especially with biological environments; however, a number of photosensitized processes, including photodynamic therapy (PDT),^3^ or photocatalytic uncaging reactions,^4^ have been demonstrated to work under biorelevant conditions.^5^ Nonetheless, the photocatalytic bond-forming assembly of desired products in cellular environments remains to be demonstrated.^6^ Adding this type of transformations to the toolbox of life compatible synthetic reactions^7,8^ might considerably broaden this field of abiotic biological chemistry.

**Figure 1:**
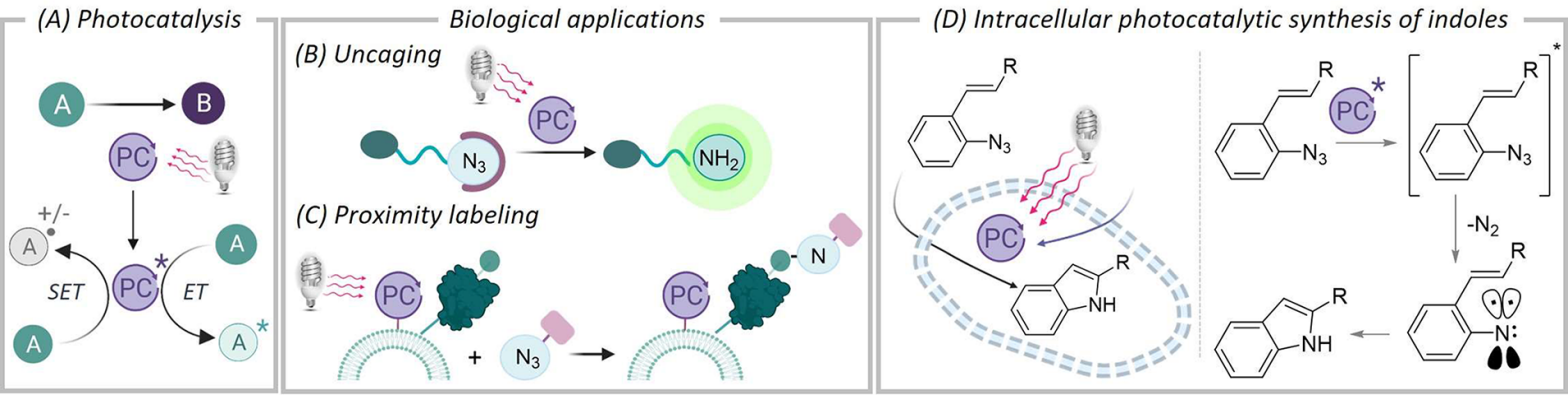
(A) Outline of direct photocatalytic activation mechanisms. (B and C) Schematic representation of photobiological applications of aryl azides. (D) This work: Photocatalytic synthesis of indoles in cells and schematic outline of a putative mechanism.

Among the different functional groups that have been harnessed for photobiological applications, aryl azides occupy a prominent position.^9^ These groups can be directly photolyzed with high-energy light, but this type of irradiation is not adequate for biological uses. Fortunately, they can also be activated at longer wavelengths using appropriate photocatalysts.^10^ Indeed, the photocatalytic reduction of aryl azides to the corresponding amines has been performed in cellular contexts (Figure 1B).^11^ Aryl azides can also be converted into aryl nitrene or aminyl radical species, which can react and label nearby proteins (Figure 1C).^12^

On these grounds, we questioned whether the photocatalytic conversion of aryl azides into nitrene intermediates could be harnessed to perform synthetic reactions inside living mammalian cells. Herein, we present compelling evidence for the feasibility of this idea. Specifically, we report the intracellular synthesis of 2-substituted indoles from *ortho*-azido styrylarenes, using visible-light photocatalysis (Figure 1D). This type of annulation is known to work in organic solvents, and has been invoked to involve nitrene intermediates generated via energy transfer mechanisms, but had never been tested in aqueous or biorelevant media.^13^

We selected an intramolecular reaction not only to maximize bioorthogonality and favor the desired C(sp^2^)–H amination over alternative reactivities of the putative nitrene intermediate but also because indoles are privileged structures in terms of biological potential. The viability of the approach was studied with aryl azide **1a**, previously shown to provide the indole when irradiated with white light in the presence of a ruthenium polypyridyl photocatalyst.^13^ The reaction was described in organic solvents at high concentrations (>50 mM), conditions that are far away from those required for the intended biological use.

Irradiation (blue LED lamp) of a 50 mM solution of **1a** in degassed DMF, for 3 h under Ar, in the presence of 2 mol % of [Ru(bpy)_3_][PF_6_]_2_ (from now on Ru(bpy)_3_), resulted in the clean production of 2-phenylindole **2a** in 94% yield (Table 1, entry 1). Similar results were obtained in MeCN or DMSO (entries 2, 3). Switching the solvent to a 1:1 mixture of water/DMF gave the product in 87% yield (entry 5). Higher proportions of water (8:2) brought lower efficiencies (entry 6); but with 5 mol % of the catalyst, **2a** was obtained in 77% yield (10 mM, entry 7). The reaction was also very efficient at 1 mM, irradiating for only 10 min, and even at 250 μM (entries 8–10). In the dark, with or without the Ru photocatalyst, there is no conversion (entries 11, 12). However, direct irradiation of a solution of the substrate produces a 22% yield of **2a** (10 min, entry 13).

**Table 1:**
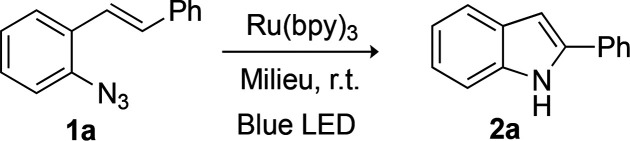

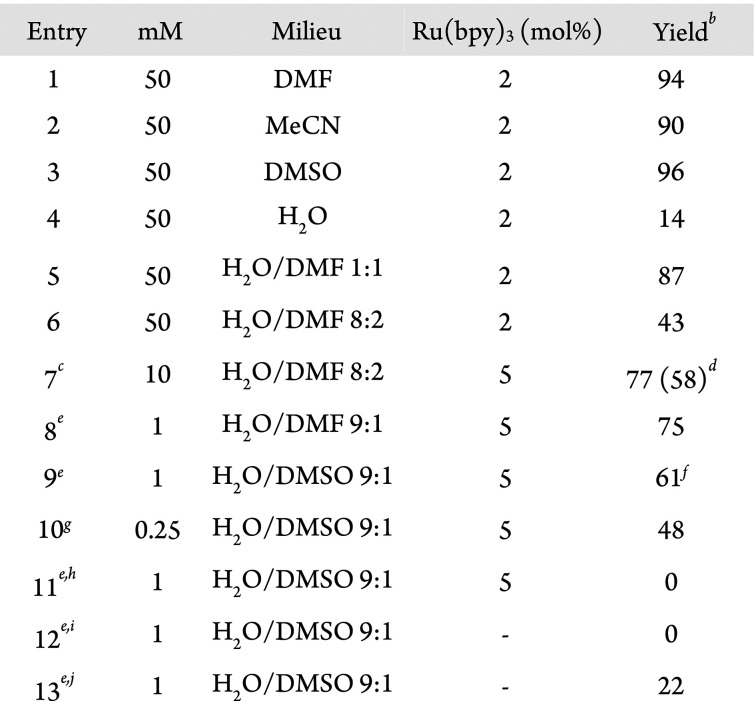
Photocatalyzed Reaction under Aqueous Conditions^a^

a Initial conditions: **1a** (0.25 mmol), Ru(bpy)_3_ (2 mol %), deoxygenated milieu (5 mL, 50 mM scale), blue LED (200 mW cm^–2^, 40 W, λ_max_ = 456 nm, 3 h).

b Yield determined by ^1^H NMR using 1,3,5-trimethoxybenzene as internal standard.

c Conditions: **1a** (0.05 mmol), Ru(bpy)_3_ (5 mol %), milieu (5 mL, 10 mM).

d Isolated yield.

e Conditions: **1a** (5 μmol), milieu (5 mL, 1 mM), 10 min irradiation.

f Experiment performed in an air-open flask.

g Conditions: **1a** (1.25 μmol), milieu (5 mL, 250 μM).

h No irradiation.

i No photocatalyst, no irradiation.

j No photocatalyst, only irradiation.

The photocatalytic reaction can also be efficiently carried out in a 1:9 mixture of DMSO/PBS (1 mM, pH 7.4), using 10 mol % of the catalyst (63% yield, Figure S1 and Table S1), and it is slightly less efficient when PBS was replaced by cell culture media such as DMEM (Dulbecco’s media, 38% yield), or HEPES (55% yield). Importantly, the process is also compatible with HeLa cell lysates (66% yield) and whole cell suspensions (80% yield). In the absence of the photocatalyst, under irradiation, yields were lower than 20%. Intriguingly, the reaction is compatible with 1 equiv of GSH, NADH, or ascorbate (Figure S1 and Tables S2–S3), as well as with BSA.

These results encouraged us to move to live cell settings. The initial protocol consisted of treating HeLa cell cultures with substrate **1a** (50 μM, 150 nmol) and the photocatalyst (50 μM) for 15 min, followed by two washing steps with PBS to eliminate excess of reactants, and irradiation of the plates in HEPES-DMEM for 45 min (Figure 2A). The cellular content was then extracted with acetonitrile (3×) and analyzed by LC-MS (Section S11).^14^ This analysis corroborated the presence of the indole **2a**, together with unreacted azide (Figure 2B,C), both in much higher proportion in the cell extracts than in the extracellular media. We could even estimate the amount of product formed intracellularly (around 4.5 nmol, 1.1 nmol/10^6^ cells, Table S5).

**Figure 2:**
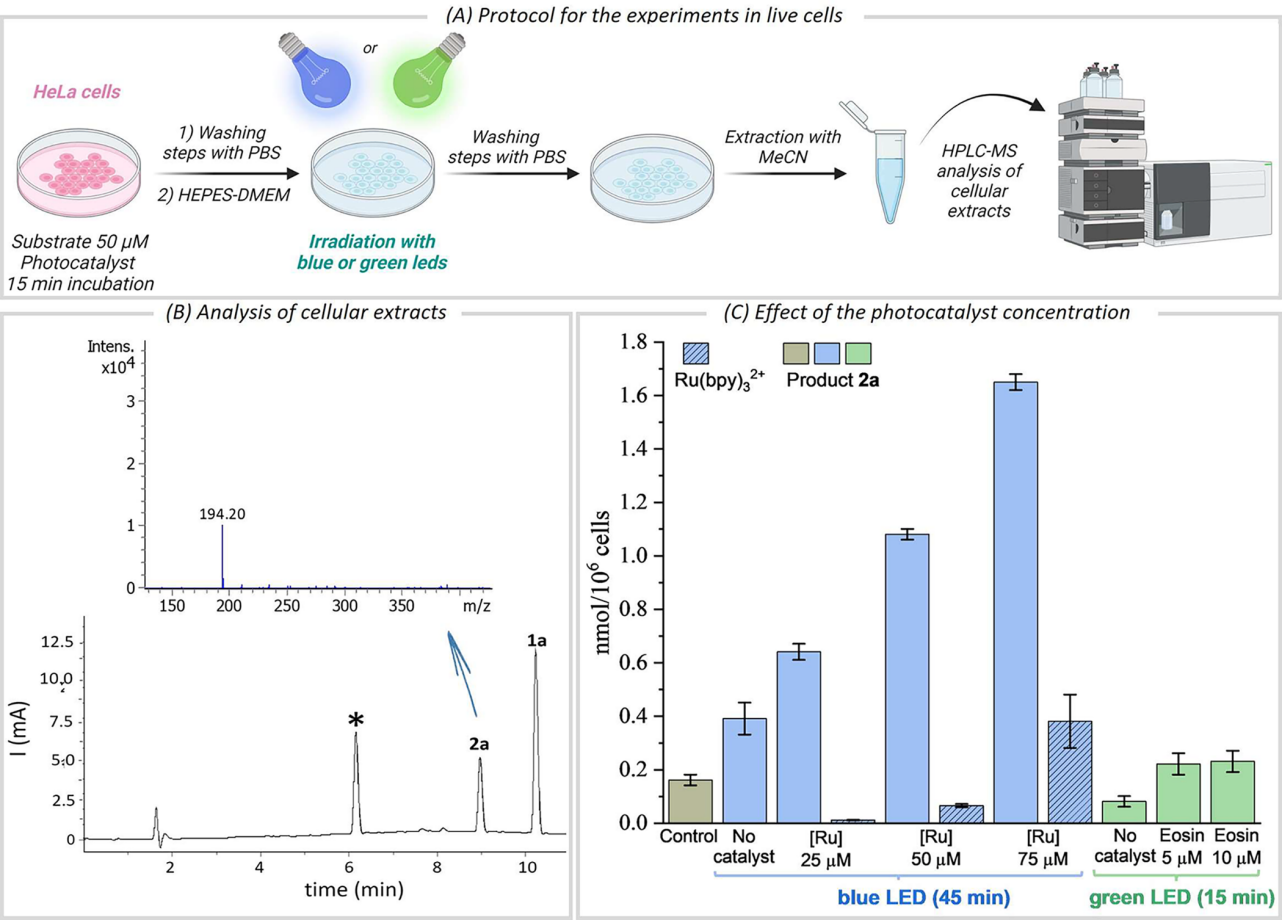
Reaction in HeLa cell cultures. (A) Experimental protocol. (B) Detection and quantification of **2a** (acetonitrile extract) for the experiment with [Ru] = 50 μM (chromatogram and inset with the mass peak of **2a**. (C) Quantification of the intracellular product **2a** (gray, blue and green bars) and Ru(bpy)_3_ (dashed blue bars). Control = Cells treated with 50 μM of **1a**, no irradiation, and no Ru(bpy)_3_. Error bars: standard deviation of three experiments. Blue LEDs: 45 min of irradiation, λ_max_ = 456 nm; Green LEDs: 15 min of irradiation, λ_max_ = 525 nm (20 mW cm^–2^). * = Coumarin as internal standard; [Ru] = Ru(bpy)_3_; and Eosin = Eosin Y.

Control experiments revealed that without irradiation and photocatalyst, the product was formed just in traces (Figure 2C, Table S6). However, under irradiation, in the absence of photocatalyst, we did observe the indole **2a**, although in a low proportion (Figure 2C, Table S7). Significantly, increasing the loading of Ru(bpy)_3_ to 75 μM we detected a higher amount of intracellular product (over 1.7 nmol/10^6^ cells, Table S8), which is in consonance with the presence of more photocatalyst inside the cells. Indeed, we were able to analyze and quantify the intracellular presence of Ru(bpy)_3_ by LC/MS (Table S13), which allowed to infer a clear correlation between the amount of photocatalyst and the reaction efficiency (Figure 2C).

These results further confirm the photocatalytic nature of the reaction and unveil the existence of some catalytic turnover. Moreover, the amount of product is dependent on the number of cells used in the experiments, which together with the absence of extracellular catalyst before the irradiation (Section S11) further supports the intracellular character of the transformation. The reaction could also be performed in other cell lines such as A549 (Table S11). MTT assays confirmed that neither the substrate, the product, or the catalyst nor the irradiation generates a noticeable toxicity under the reaction conditions (Section S9).

We next wondered whether the use of less energetic light could avoid direct photoexcitation of the substrate. Indeed, *in vitro* (glass vial) reactions performed in PBS/DMSO (1 mM) with green LEDs (10 min irradiation, λ_max_ = 525 nm), without photocatalyst, led to yields lower than 10% yield. However, running the same experiment in the presence of 10 mol % of a green-shifted photosensitizer, Eosin Y, we observed a significant increase in yield (up to 34%, Table S1).

Importantly, *in cellulo* experiments confirmed a similar trend. Therefore, addition of 50 μM of **1a** to HeLa cells, and green-light irradiation of the cell culture for 15 min led to only traces of product **2a**. In contrast, when the same experiment was carried out in cells preincubated with 10 μM of Eosin Y, we detected a small but meaningful increase in the amount of indole **2a** inside the cells (Figure 2C, green bars).

At this stage, we considered it relevant to demonstrate that the technology can be used to synthesize products that exhibit specific biological or physical properties; in particular, we were attracted by the possibility of building fluorescent products. Gratifyingly, irradiation of azide **1b** with blue light in the presence of Ru(bpy)_3_ (10 mol %) in PBS/DMSO 9:1 led to the fluorescent indole **2b** (54% yield, after 10 min). The reaction without a photocatalyst was also rather efficient (45% yield), likely because the substrate exhibits a slight, blue-shifted absorption with respect to **1a**. Importantly, using green light (525 nm), in the absence of photocatalyst (in PBS/DMSO 9:1, 1 mM), the reaction gave only traces of the product. However, in the presence of 10 mol % Eosin Y we observed 25% indole after 10 min of irradiation (Table S4).

With these promising results, we moved to cellular contexts. First, we confirmed that addition of product **2b** to HeLa cells induces a clear intracellular fluorescence, which was not observed with precursor **1b** (Figure 3A, panels a, b and Figure S25). The reactions were carried out by treatment of HeLa cells with 50 μM of substrate **1b** and different concentrations of Eosin Y (0–20 μM) for 15 min, followed by two washing steps with HEPES-DMEM, and irradiating the cell cultures with green light for another 15 min. Fluorescence microscopy confirmed a very clear blue signal in the cytoplasm corresponding to product **2b** (Figure 3B, panels d–f and Figures S25–S27).

**Figure 3:**
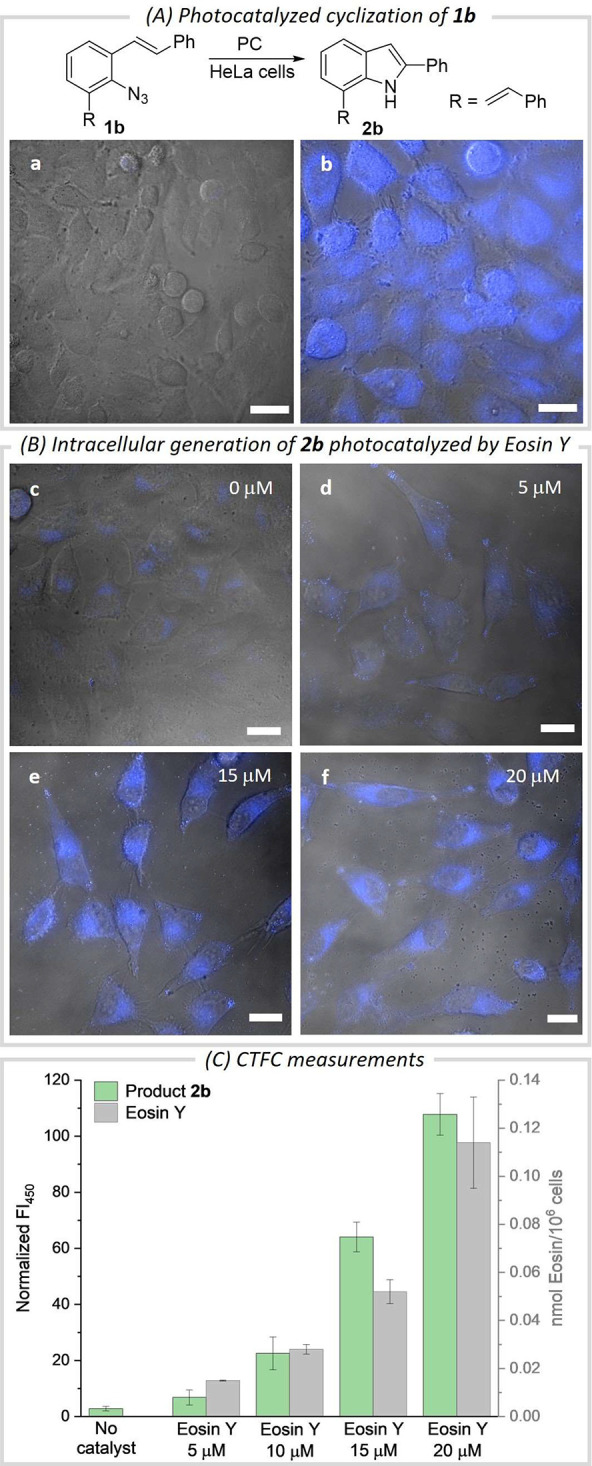
Generation of **2b** in HeLa cell cultures. (A) Photocatalytic reaction and micrographies of cells after incubation with the substrate (**1b**, a) or the product (**2b**, b). Scale bar: 20 μm. λ_exc_ = 405 nm, λ_em_ = 420–480 nm. (B) Fluorescence micrographies in reactions with increasing concentrations of Eosin Y (c–f, from 0 to 20 μM). C) Bar graphic based on CTFC measurements. The intracellular concentration of Eosin Y is also shown (gray bars). Error bars: standard deviation of three experiments. Green LEDs: 20 mW cm^–2^ (40 W lamp), λ_max_ = 525 nm, 15 min of irradiation. PC = photocatalyst.

Gratifyingly, in the absence of light, there is no fluorescence; and under irradiation, in the absence of the photosensitizer, the fluorescence output was also rather residual (Figure 3B, panel c). LC-MS analysis confirmed that under these control conditions the product **2b** is just formed in traces, while in the photocatalytic process we estimated over 0.45 nmol/10^6^ cells, when using 20 μM Eosin Y (Figure 3C and Tables S15–S16).

Importantly, we also managed to measure and quantify the amount of intracellular Eosin Y (Section S11, Figure S24 and Tables S18–S19), which revealed again a correlation between the photocatalyst concentration, and the amount of product so far formed (Figure 3C).

Finally, we questioned whether, capitalizing on the different levels of integrins expressed by cells in their surfaces,^15^ it would be possible to develop cell-selective photocatalytic reactions. Gratifyingly, we found that by using as photocatalyst the newly synthesized Eosin Y derivative **Eosin-CRGD** (Figure 4A), the fluorescence generated inside HeLa cells was considerably higher than in MCF7 cells, which contain much lower levels of integrins (Figure 4B, panels b and d).

**Figure 4:**
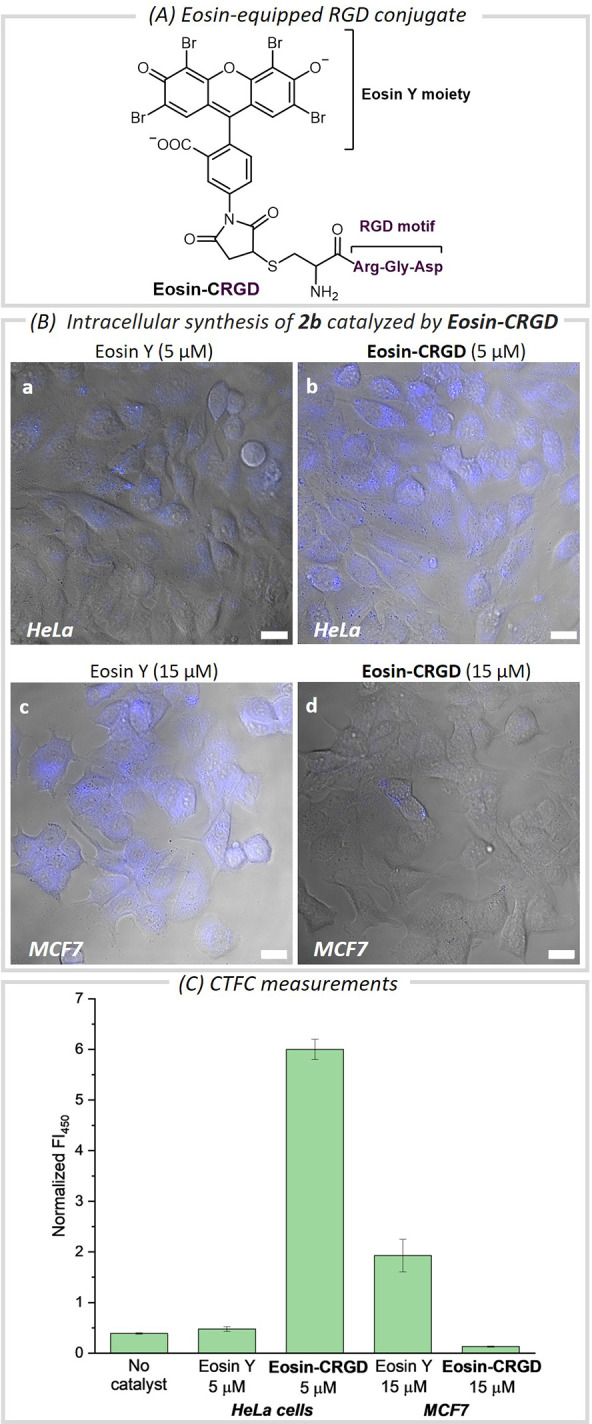
Selective cellular targeting using **Eosin-CRGD** as photocatalyst (Section S7). (A) Structure of the synthetic RGD derivative containing Eosin Y. (B) Fluorescence micrographies of HeLa (a,b) and MCF7 cells (c,d) (blue channel and brightfield) after incubation with **1b**, and Eosin Y (a,c) or **Eosin-CRGD** (b,d), and irradiation. (C) Bar graphic showing the CTFC. Reaction conditions: Cells were pretreated with 50 μM of **1b** and Eosin Y (5 μM, a), (15 μM, c) or **Eosin-CRGD** (5 μM, b), (15 μM, d), in DMEM for 15 min, washed with PBS (2×), and irradiated with green light in HEPES-DMEM for 15 min. The error bars indicated the standard deviation of three experiments. Scale bar: 20 μm. λ_exc_ = 405 nm, λ_em_ = 420–480 nm.

These results confirm that the technology may enable the selective imaging of a specific population of cancer cells and hint at a potential future application in precision medicine.

In conclusion, we have presented here the first examples of a bond-forming, photocatalytic intracellular reaction that converts exogenous substrates into valuable synthetic products. We demonstrate that an appropriate matching of excitation sources and photocatalysts allows us to considerably improve the ratio of catalyzed versus the noncatalyzed photochemical process. While our results can be regarded as proof-of-concept examples, we anticipate substantial potential for this emerging field of bioorthogonal synthetic photocatalysis.

## Supporting Information Available

Detailed procedures, materials, compound characterization, spectroscopic, NMR, HPLC data and supporting experimental data and figures (PDF)
