# Supplementary material for: Intracellular Synthesis of Indoles Enabled by Visible-Light Photocatalysis
Source: J Am Chem Soc. 2024 Jan 26;146(5):2895–900. doi: 10.1021/jacs.3c13647 (PMC10859955; doi:10.1021/jacs.3c13647)
Supplement: Supplementary file 1 — ja3c13647_si_001.pdf [file ja3c13647_si_001.pdf]

SUPPORTING INFORMATION

Intracellular Synthesis of Indoles enabled by Visible-Light Photocatalysis

Cinzia D'Avino, Sara Gutiérrez, Max J. Feldhaus, María Tomás-Gamasa,* José Luis Mascareñas*

Centro Singular de Investigación en Química Biolóxica e Materiais Moleculares (CiQUS), and Departamento de Química Orgánica. Universidade de Santiago de Compostela, 15705 Santiago de Compostela, Spain

TABLE OF CONTENTS

S1. General 2

S2. Reaction set-up for the photocatalytic transformations in vials 4

S3. Synthesis of azide precursors (1a and 1b) 4

S4. Synthesis of indoles (2a and 2b) in organic solvents 7

S5. Photocatalytic reactions in aqueous media 8

S6. Spectroscopic studies 14

S7. Synthesis of conjugate Eosin-CRGD 15

S8. General information for the cellular experiments 18

S9. Cell viability 19

S10. Photocatalytic reactions in cell cultures 22

S11. Quantification studies of experiments with cells using LC/MS 22

S12. Detection of indole derivative 2b by fluorescence microscopy 36

S13. NMR Spectra 39

S14. References 43

S1. General

Synthetic procedures for the preparation of precursors were carried out under an atmosphere of nitrogen using a vacuum-line, unless otherwise indicated. Dry solvents were directly purchased from Sigma Aldrich and used without further purification. Water was deionized and purified on a *Millipore Milli-Q® Integral system*. Phosphate buffered saline (PBS 10X, pH 7.2) was prepared following standard procedures and diluted ten times with water before use. Dulbecco's Modified Eagle's Medium (GibcoTM DMEM) was purchased from *ThermoFisher Scientific*. Fetal Bovine Serum (FBS) was purchased from *Sigma Aldrich*. HeLa cell lysates were obtained from 2 days cultured HeLa cells: after two washings with PBS, cells were scraped from the well, sonicated and diluted with DMEM to reach the indicated concentration.

Chemicals were purchased from *Sigma Aldrich, Alfa Aesar, Fluka, TCl Chemicals, Chemosapiens BLD* or *Acros Organics* and used without further purification. BSA was purchased from Sigma Aldrich as lyophilized powder with purity ≥98% (SKU-A3059).

Azide **1a** is a known compound and was synthesized according to literature procedures.[1] The ^{1}H and ^{13}C NMR data were in complete agreement with the reported values.

Reactions with the azide precursors were carried out under an atmosphere of argon using vacuum-line, unless otherwise indicated. Reaction mixtures were stirred using Teflon-coated magnetic stir bars. The abbreviation "rt" refers to reactions carried out approximately at 23 °C. Temperature was maintained using Thermowatch-controlled heating blocks. Thin layer chromatography (TLC) was performed on silica gel plates (*Merck 60* silica gel F_{254}) plates and components were visualized by observation under UV and / or by using staining solutions of $KMnO_4$ or *p*-anisaldehyde and heating. Chromatographic purification of products was accomplished using flash column chromatography on silica gel (*Merck Geduran®* Si 60, 40 – 63 µm, silica gel, normal phase) or by reversed-phase high-performance liquid chromatography (RP-HPLC).

Concentration refers to the removal of volatile solvents via distillation using a rotary evaporator Buchi R-210 equipped with a thermostated bath B-491, a vacuum regulator V-850, and a vacuum pump V700, followed by residual solvent removal under high vacuum. Dryings were performed with anhydrous Na_2SO_4.

^{1}H NMR (300 MHz) and ^{13}C NMR (75 MHz) spectra were recorded at room temperature on a *Varian Mercury* 300 MHz spectrometer. ^{1}H NMR (500 MHz) and ^{13}C NMR (126 MHz) spectra were

recorded at room temperature on a *Bruker DRX-500* spectrometer. Multiplicities are abbreviated as follows: s = singlet, d = doublet, t = triplet, q = quartet, quint = quintet, m = multiplet, br = broad and combinations of these. The coupling constants (*J*) are given in Hz. The chemical shifts for protons (δ) are reported in parts per million (ppm) downfield from tetramethylsilane and are referenced to residual protium in the NMR solvent ($CDCl_3$ δ = 7.26, CD_2Cl_2 δ = 5.32). Chemical shifts for carbon are reported in parts per million downfield from tetramethylsilane and are referenced to the carbon resonances of the solvent ($CDCl_3$ δ = 77.2, CD_2Cl_2 δ = 53.8). NMR spectra were analyzed using *MestreNova©* NMR data processing software (www.mestrelab.com).

UV Measurements were performed using a Jasco V-770 spectrometer.

Fluorescence measurements were performed using an *Edinburgh FS5 spectrofluorometer* thermostated cell compartment at 20 ± 0.5 °C using 1 cm quartz cells. The measurements were made with the following settings: increment 0.5 nm, averaging time 0.1 s, excitation slit width 1.5 nm, emission slit width 1.5 nm.

Analytical HPLC of the reactions with **1a** were performed on an *Agilent 1260 Infinity II* coupled to an *Agilent Technologies 6120 Quadrupole LC-MS* using a flow rate of 0.35 mL/min at room temperature. The initial conditions for the solvent system were H_2O/MeCN (95:5) followed by a gradual change over 12 min to H_2O/MeCN (5:95). The chromatogram was recorded using a UV detection at λ = 310 nm. HPLC analysis were also performed on a *THERMO Ultimate 3000* coupled to a *Bruker AmaZon SL ion trap LC-MS* using a flow rate of 0.35 mL/min at room temperature. The initial conditions for the solvent system were H_2O/MeCN (80:20) followed by a step directly at 5 min to H_2O/MeCN (30:70), at 9 min to H_2O/MeCN (5:95), and a gradual change over 13 min to H_2O/MeCN (80:20). The chromatogram was recorded using a UV detection at λ = 300 nm.

Quantification experiments by HPLC-MS were performed on *THERMO Ultimate 3000* or *Agilent 1260 Infinity II.*

In vitro (reactions in glass vessels) photocatalytic experiments with blue or green light were performed using a Kessil LEDs 40 W (PR160 – either at 456 nm or 525 nm). For full details see on their emission properties, see: https://kessil.com/. Lamps were placed at approximately 4 cm from the reaction vessel. Temperature was controlled with a cooling fan and kept in a range of 20-25 °C).

S2. Reaction set-up for the photocatalytic transformations in vials

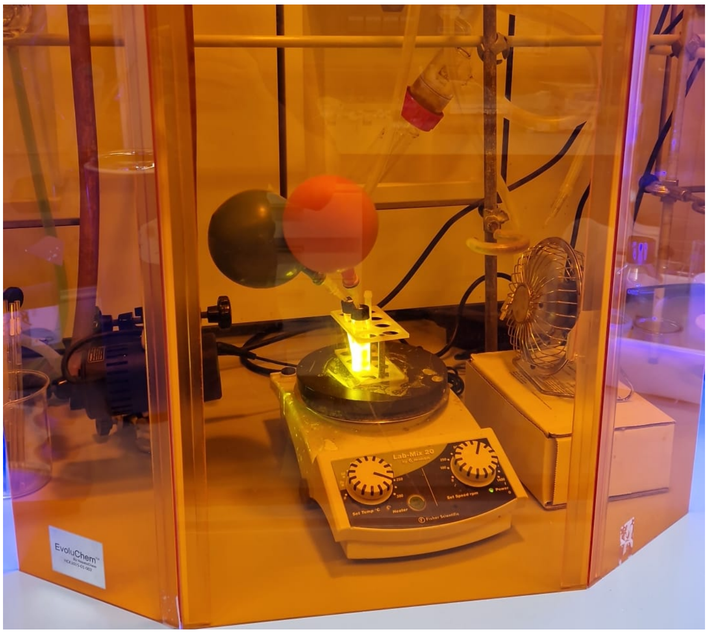

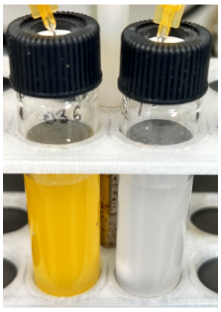

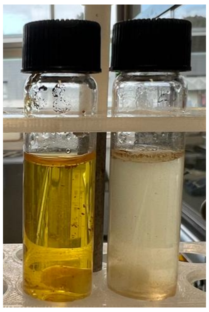

Illustration S1. *Pictures of the "reaction set-up" using Kessil Blue LED with cooling fans (up). Crude of the reaction of substrate **1a** with photocatalyst (Ru(bpy)$_3$, orange) and w/o photocatalyst (white) before (down left) and after 10 min of irradiation (down right) in PBS/DMSO (9:1).*

S3. Synthesis of azide precursors (1a and 1b)

Synthesis of (*E*)-2-styrylaniline

Procedure adapted from Xia et *al.*[1]

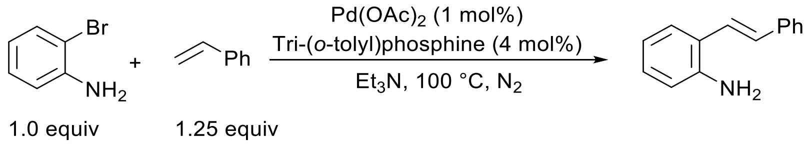

A mixture of substituted 2-bromoaniline (10 mmol, 1.0 equiv, 1.1 mL), styrene (12.5 mmol, 1.25 equiv, 1.4 mL), Pd(OAc)$_2$ (0.1 mmol, 0.01 equiv, 22.5 mg), tri-(*o*-tolyl)phosphine (0.4 mmol, 0.04 equiv, 121.7 mg), and triethylamine (10 mL) was heated to 100 °C in a nitrogen filled Schlenk

tube. The completion of the reaction was monitored by TLC by the consumption of substituted 2-bromoaniline (2 h), and then the system was cooled down to room temperature.

The cooled mixture was broken-up in Et_2O (30 mL) and filtered. The amine salts were washed with Et_2O. The combined organics were concentrated and purified by silica flash chromatography using hexane/EtOAc (9:1) as eluent to afford product (*E*)-2-styrylaniline as a white solid. ^{1}H NMR in concordance with the data reported in literature.[1]

R_f = 0.26 (Hexane/EtOAc 9:1)

Yield = 86% (8.6 mmol, 1.68 g)

^{1}H-NMR (300 MHz, $CDCl_3$): δ (ppm) 7.55–7.48 (m, 2H), 7.42 (dd, *J* = 7.7, 1.5 Hz, 1H), 7.36 (t, *J* = 7.5 Hz, 2H), 7.25 (dd, *J* = 13.1, 6.2 Hz, 1H), 7.20 – 7.07 (m, 2H), 7.00 (d, *J* = 16.1 Hz, 1H), 6.84 (t, *J* = 7.5 Hz, 1H), 6.76 (d, *J* = 7.9 Hz, 1H), 4.18 (br, 2H)

Synthesis of (*E*)-1-azido-2-styrylbenzene (1a)

Procedure adapted from Xia et *al.*[1]

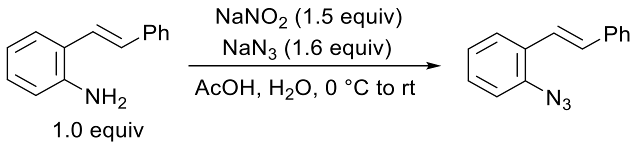

To a cooled mixture (0 °C) of substituted styryl aniline (8.55 mmol, 1.0 equiv, 1.67 g) in AcOH (48 mL) and H_2O (48 mL) was added $NaNO_2$ (12.83 mmol, 1.5 equiv, 885 mg). After 1 h, NaN_3 (13.68 mmol, 1.6 equiv, 890 mg) was added portion wise to the above mixture, then the system was allowed to warm to room temperature. After 0.5 h, the mixture was diluted with Et_2O (48 mL) and H_2O (48 mL). Na_2CO_3 was slowly added until pH = 7 over ice-bath. The aqueous phase was extracted with Et_2O (100 mL x 3). The organic phases were combined and washed with H_2O (100 mL) and brine (100 mL) and dried over $MgSO_4$. The styryl azide was purified by flash chromatography on silica gel (gradient from hexane to hexane/EtOAc 95:5) to afford product **1a** as a light-yellow solid.^{1}H NMR in concordance with the data reported in literature.[1]

R_f = 0.47 (Hexane/EtOAc 95:5)

Yield = 91% (7.8 mmol, 1.73 g)

^{1}H NMR (300 MHz, CDCl3): δ (ppm) 7.64 (dd, *J* = 7.8, 1.5 Hz, 1H), 7.58 - 7.48 (m, 2H), 7.41 – 7.31 (m, 3H), 7.31 – 7.23 (m, 2H), 7.20 – 7.13 (m, 2H), 7.06 (s, 1H)

Synthesis of 2,6-di-[(*E*)-styryl]aniline

Procedure adapted from Xia et *al.*[1]

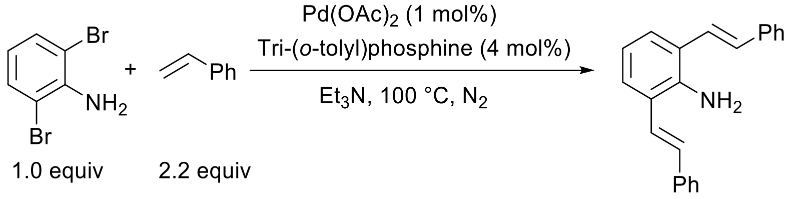

A mixture of substituted 2,6-dibromoaniline (5 mmol,1.0 equiv, 1.25 g), styrene (11.0 mmol, 2.2 equiv, 1.3 mL), $Pd(OAc)_2$ (0.05 mmol, 0.01 equiv, 11.2 mg), tri-(*o*-tolyl)phosphine (0.2 mmol, 0.04 equiv, 60.9 mg), and triethylamine (5 mL) were heated to 100 °C in a nitrogen filled Schlenk tube. The completion of the reaction was monitored by TLC by the consumption of substituted 2,6-dibromoaniline (overnight), and then the system was cooled down to room temperature.

The cooled mixture was broken-up in Et_2O (15 mL) and filtered. The amine salts were washed with Et_2O. The combined organics were concentrated and purified by silica flash chromatography using hexane/EtOAc (97:3) as eluent to afford product 2,6-di[(*E*)-styryl]aniline as a yellow solid.

$\mathbf{R_f}$ = 0.21 (Hexane/EtOAc 9:1)

Yield = 52% (2.57 mmol, 765 mg)

^{1}H-NMR (300 MHz, $CDCl_3$): δ (ppm) 7.56–7.49 (m, 4H), 7.42-7.32 (m, 6H), 7.31-7.24 (m, 2H), 7.19 (d, *J* = 16.1 Hz, 2H), 7.00 (d, *J* = 16.0 Hz, 2H), 6.84 (t, *J* = 7.6 Hz, 1H), 4.03 (br, 2H)

^{13}C NMR (75 MHz, $CDCl_3$): δ (ppm) 137.7, 131.5, 128.9 (x2), 127.9, 127.2, 126.6 (x2), 124.7, 124.6, 119.1

ESI/MS (m/z): Calculated for $C_{22}H_{19}N$ $[M+H]^+$ = 298.15; found: 298.01 $[M+H]^+$

Synthesis of [(1*E*,1'*E*)-(2-azido-1,3-phenylene)bis(ethene-2,1-diyl)]dibenzene (1b)

Procedure adapted from Barral et *al.*[2]

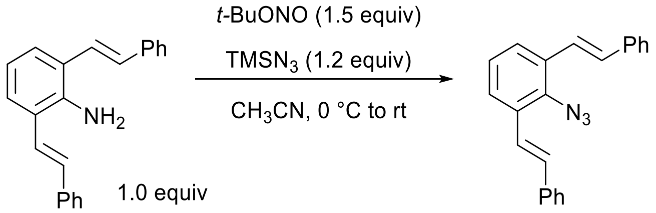

Substituted styryl aniline (2.0 mmol, 1.0 equiv, 594 mg) was dissolved in CH_3CN (4 mL) in a 25 mL round bottomed flask and cooled to 0 °C in an ice bath. To this stirred mixture was added *t*-BuONO (3.0 mmol, 1.5 equiv, 356 μL) followed by $TMSN_3$ (2.4 mmol, 1.2 equiv, 318 μL) dropwise. The resulting solution was stirred at room temperature for 5 h. The styryl azide was concentrated under vacuum and purified by silica gel chromatography (hexane/EtOAc 97:3 with a 10% of toluene) to give the product **1b** as a pale-yellow powder. **1b** is obtained as 99% of isomer E.

$\mathbf{R_f}$ = 0.49 (Hexane/EtOAc 9:1)

Yield = 69% (1.4 mmol, 443 mg)

^{1}H NMR (500 MHz, CDCl3): δ (ppm) 7.58 (dd, *J* = 15.3, 7.7 Hz, 7H), 7.48 (d, *J* = 16.3 Hz, 2H), 7.44 – 7.39 (m, 3H), 7.36 – 7.29 (m, 2H), 7.29 – 7.22 (m, 1H), 7.11 (d, *J* = 16.2 Hz, 2H)

^{13}C NMR (126 MHz, $CDCl_3$): δ (ppm) 137.3, 135.3, 132.8, 132.0, 128.9 (x2), 128.2, 126.9 (x2), 126.4, 126.2, 123.7

ESI/MS (m/z): Calculated for $C_{22}H_{17}N_3$ $[M-N_2+H]^+$ = 296.14; found: 296.22 $[M-N_2+H]^+$

S4. Synthesis of indoles (2a and 2b) in organic solvents

Representative procedure for the synthesis of indole 2a

This protocol corresponds to entry 1 in Table 1.[1]

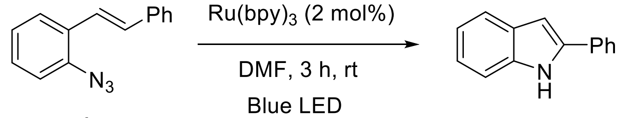

To a 5 mL vial equipped with magnetic stirrer bar was added **1a** (0.1 mmol, 22.1 mg) and $Ru(bpy)_3$ (0.002 mmol, 0.02 equiv, 1.7 mg). Several vacuum/nitrogen cycles were performed, and the vial was filled with Argon. Then, 2 mL of deoxygenated DMF were added. The reaction was initiated with the irradiation of a Kessil 40 W blue lamp and allowed to react for 3 h under fan refrigeration. The crude was transferred to a separating funnel and extracted with Et_2O (10 mL x 3). The combined organic phases were dried over anhydrous Na_2SO_4, filtered, and concentrated under reduced pressure. The indole was purified by silica gel chromatography (hexane/EtOAc 9:1) to give the product 2-phenyl-1H-indole **2a** as a yellow solid.

$\mathbf{R_f}$ = 0.61 (Hexane/EtOAc 9:1)

Yield = 94% (0.094 mmol, 18.2 mg)

^{1}H-NMR (300 MHz, $CDCl_3$): δ (ppm) 8.34 (s, 1H), 7.71 – 7.60 (m, 3H), 7.49 – 7.37 (m, 3H), 7.37 – 7.29 (m, 1H), 7.23 – 7.09 (m, 2H), 6.83 (dd, *J* = 2.1, 0.9 Hz, 1H)

Representative procedure for the synthesis of indole 2b

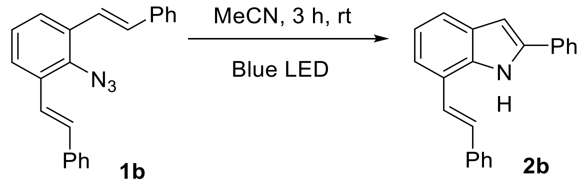

To a 5 mL vial equipped with magnetic stirrer bar was added **1b** (0.155 mmol, 50.0 mg). Several vacuum/nitrogen cycles were performed, and the vial was filled with Argon. Then, 3.1 mL of deoxygenated MeCN were added. The reaction was initiated with the irradiation of a Kessil 40 W blue lamp and allowed to react for 3 h under fan refrigeration. The crude was concentrated under reduced pressure and purified by silica gel chromatography (hexane/toluene 2:1) to give the product *(E)*-2-phenyl-7-styryl-1H-indole **2b** as a white solid.

$\mathbf{R_f}$ = 0.34 (Hexane/EtOAc 9:1)

Yield = 62% (0.096 mmol, 28.4 mg)

^{1}H-NMR (500 MHz, CD_2Cl_2): δ (ppm) 8.70 (s, 1H), 7.78 – 7.72 (m, 3H), 7.65 – 7.61 (m, 3H), 7.59 – 7.55 (m, 1H), 7.51 – 7.45 (m, 4H), 7.44 – 7.39 (m, 4H), 7.39 – 7.34 (m, 1H), 7.33 – 7.29 (m, 1H), 7.27 (d, *J* = 16.3 Hz, 1H), 7.15 (t, *J* = 7.6 Hz, 2H), 6.89 (d, *J* = 2.1 Hz, 1H)

S5. Photocatalytic reactions in aqueous media

Representative procedure for the synthesis of indole 2a in H_2O/DMF

This protocol corresponds to entry 7 in Table 1 of the main manuscript

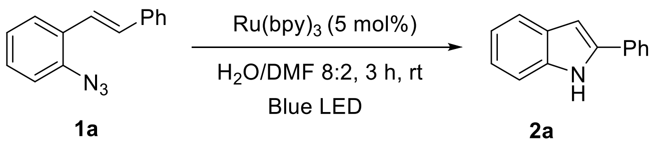

To a 5 mL vial equipped with magnetic stirrer bar was added **1a** (0.05 mmol, 11.1 mg, 10 mM) and Ru(bpy)$_3$ (0.0025 mmol, 0.05 equiv, 2.2 mg). Several vacuum/nitrogen cycles were performed, and the vial was filled with Argon. Then, 1 mL of deoxygenated DMF and 4 mL of deoxygenated H_2O were added. The reaction was initiated with the irradiation of a Kessil 40 W

blue lamp (456 nm, 200 mW cm^{-2}, 40 W, situated at ≈ 4 cm from the reaction vessel) and allowed to react for 3 h under fan refrigeration. The crude was transferred to a separating funnel and extracted with Et_2O (10 mL x 3). The combined organic phases were dried over anhydrous Na_2SO_4, filtered, and concentrated under reduced pressure. The crude yield was determined by ^1H NMR using 1,3,5-trimethoxy-benzene (TMB) as internal standard. After, to compare the yield, the indole **2a** was purified by silica column (hexane/EtOAc 9:1).

Yield calculated in the crude with internal standard = 77% (0.039 mmol, 7.4 mg)

Yield of the product isolated by silica column = 58% (0.029 mmol, 5.6 mg)

Representative procedure for the synthesis of indole 2a in PBS/DMSO

This protocol corresponds to the reaction in PBS/DMSO (1 mM).

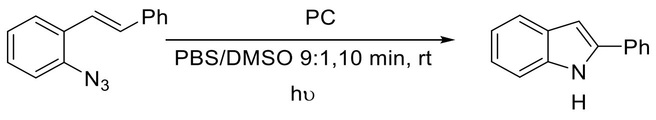

1a **2a**

To a 5 mL vial equipped with magnetic stirrer bar, several vacuum/nitrogen cycles were performed, and the vial was filled with Argon. A stock solution of **1a** (1.1 mg, 5 μmol in 250 μL of deoxygenated DMSO) was added, followed by a stock solution of the photocatalyst (PC, 0.5 μmol in 250 μL of deoxygenated DMSO, 10 mol%). Then, 4.5 mL of deoxygenated PBS were added. The reaction was initiated with the irradiation of a Kessil 40 W blue or green lamp and allowed to react for 10 min under fan refrigeration. The crude was transferred to a separating funnel and extracted with Et_2O (10 mL x 3). The combined organic phases were dried over anhydrous Na_2SO_4, filtered, and concentrated under reduced pressure. The crude yield was determined by ^1H NMR using 1,3,5-trimethoxy-benzene (TMB) as internal standard.

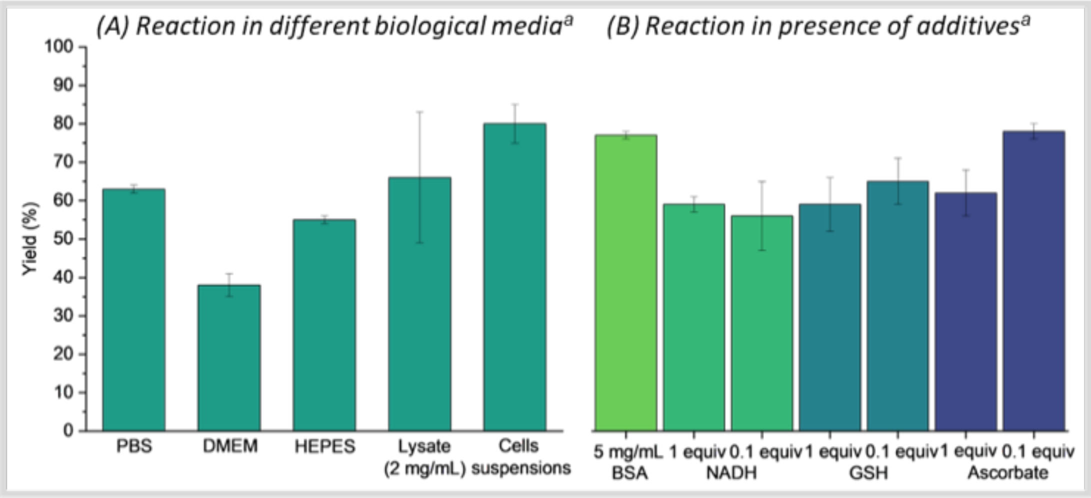

Figure S1. *Biomolecular orthogonality studies: A) Influence of biological media. B) Influence of relevant additives.* [a] ***1a*** *(5 µmol), Ru(bpy)$_3$ (10 mol%), milieu (5 mL, 1:9 DMSO/biological milieu, 1 mM), 10 min irradiation, blue LED lamp.*

Table S1. Results and control experiments.[a]

Entry	PC	Light	Yield (%)[b]
1	-	-	0
2	Ru(bpy)$_3$	-	0
3	Eosin Y	-	0
4	-	Blue	20
5	Ru(bpy)$_3$	Blue	68
6	-	Green	10
7	Eosin Y	Green	34

[a] *Conditions:* ***1a*** *(5 µmol), photocatalyst (PC, 10 mol%) if used, PBS (4.5 mL, 1 mM), DMSO (0.5 mL, 1 mM), blue LEDs (456 nm, 200 mW cm^{-2}, 40 W) or green LEDs (525 nm, 200 mW cm^{-2}, 40 W) situated at ≈ 4 cm from the reaction vessel, 10 min, rt.* [b] *Yields determined by ^{1}H NMR using 1,3,5-trimethoxybenzene (TMB) as internal standard.*

Table S2. Control experiments in the absence of photocatalyst, in different media.[a]

Entry	Milieu	Yield (%)[b]
1	PBS	19
2	DMEM	14
3	DMEM HEPES	20
4	Lysate (1.94 mg/mL)	20
5	Cells suspension	21

[a] *Conditions:* ***1a*** *(5 µmol), milieu (5 mL, 1 mM), blue LEDs (456 nm, 200 mW cm^{-2}, 40 W) situated at ≈ 4 cm from the reaction vessel, 10 min, rt.* [b] *Yields determined by ^{1}H NMR using 1,3,5-trimethoxybenzene as internal standard.*

Table S3. Control experiments in presence of additives, in the absence of photocatalyst.[a]

Entry	Biological molecule	Yield (%)[b]
1	BSA (5 mg/mL)	20
2	NADH 1 equiv	24
3	NADH 0.1 equiv	24
4	GSH 1 equiv	25
5	GSH 0.1 equiv	15
6	Ascorbate 1 equiv	28
7	Ascorbate 0.1 equiv	27

[a] *Conditions:* ***1a*** *(5 μmol, 1 equiv), PBS (4.5 mL, 1 mM), DMSO (0.5 mL, 1 mM), blue LEDs (456 nm, 200 mW cm^{-2}, 40 W) situated at ≈ 4 cm from the reaction vessel, 10 min, rt.* [b] *Yields determined by ^{1}H NMR using 1,3,5-trimethoxybenzene as internal standard.*

Representative NMR spectrum for the transformation of azide 1a into indole 2a

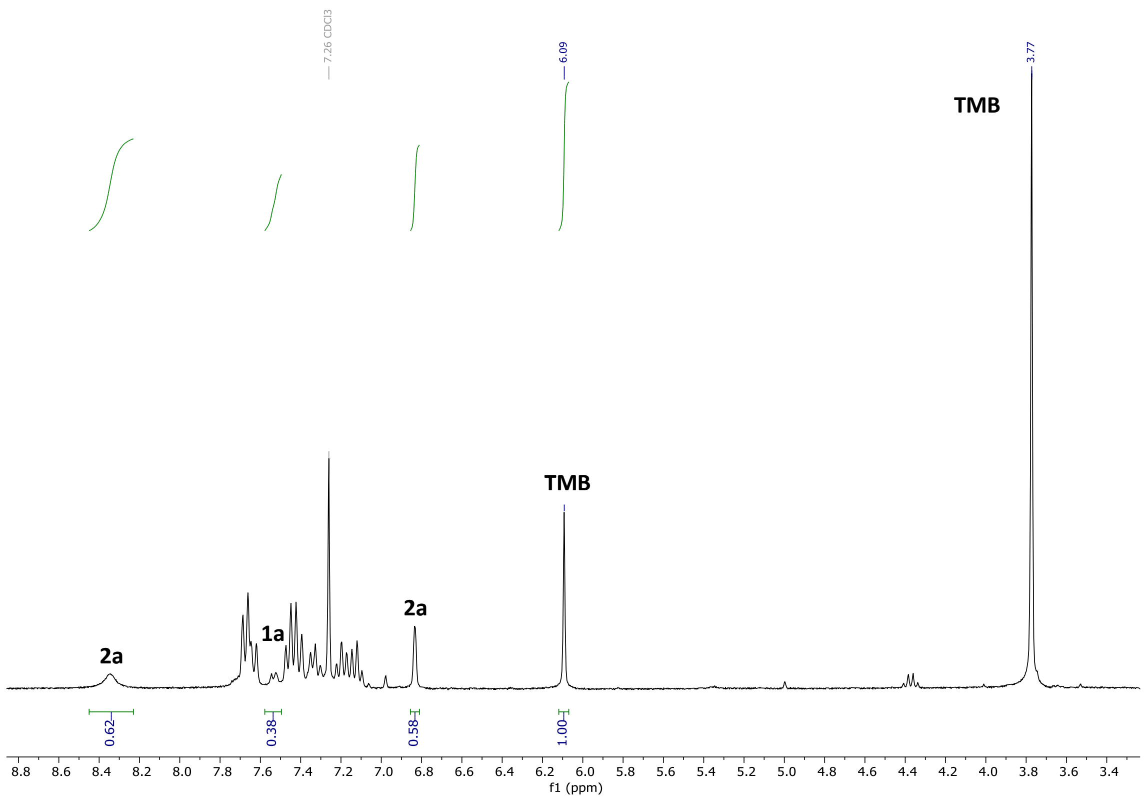

***Figure S2**. ^{1}H NMR of the crude of the reaction to produce **2a** in PBS/DMSO 9:1 with Ru(bpy)$_3$ and blue LEDs. TMB is 1,3,5-trimethoxybenzene.*

Representative general procedure for the synthesis of (*E*)-2-phenyl-7-styryl-1*H*-indole (2b) from azide 1b in aqueous media

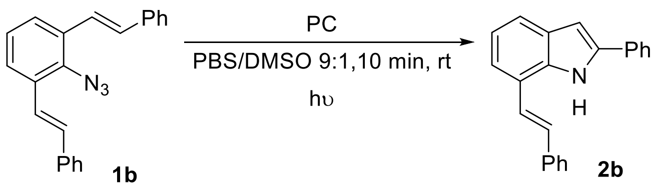

To a 5 mL vial equipped with magnetic stirrer bar vacuum/nitrogen cycles were performed, and the vial was filled with Argon. A stock solution of **1b** (1.6 mg, 5 μmol in 250 μL of deoxygenated DMSO) was added, followed by a stock solution of a photocatalyst (PC, 0.5 μmol in 250 μL of deoxygenated DMSO, 10 mol%). Then, 4.5 mL of deoxygenated PBS were added (final concentration: 1 mM). The reaction was initiated with the irradiation of a Kessil 40 W blue or green lamp and allowed to react for 10 min under fan refrigeration. The crude was transferred to a separating funnel and extracted with Et_2O (10 mL x 3). The combined organic phases were dried over anhydrous Na_2SO_4, filtered, and concentrated under reduced pressure. The crude yield was determined by ^{1}H NMR using 1,3,5-trimethoxy-benzene as internal standard.

^{1}H NMR in concordance with the data reported in the literature, and prepared using other procedure.[3]

Representative NMR spectra for the transformation of azide 1b to indole 2b

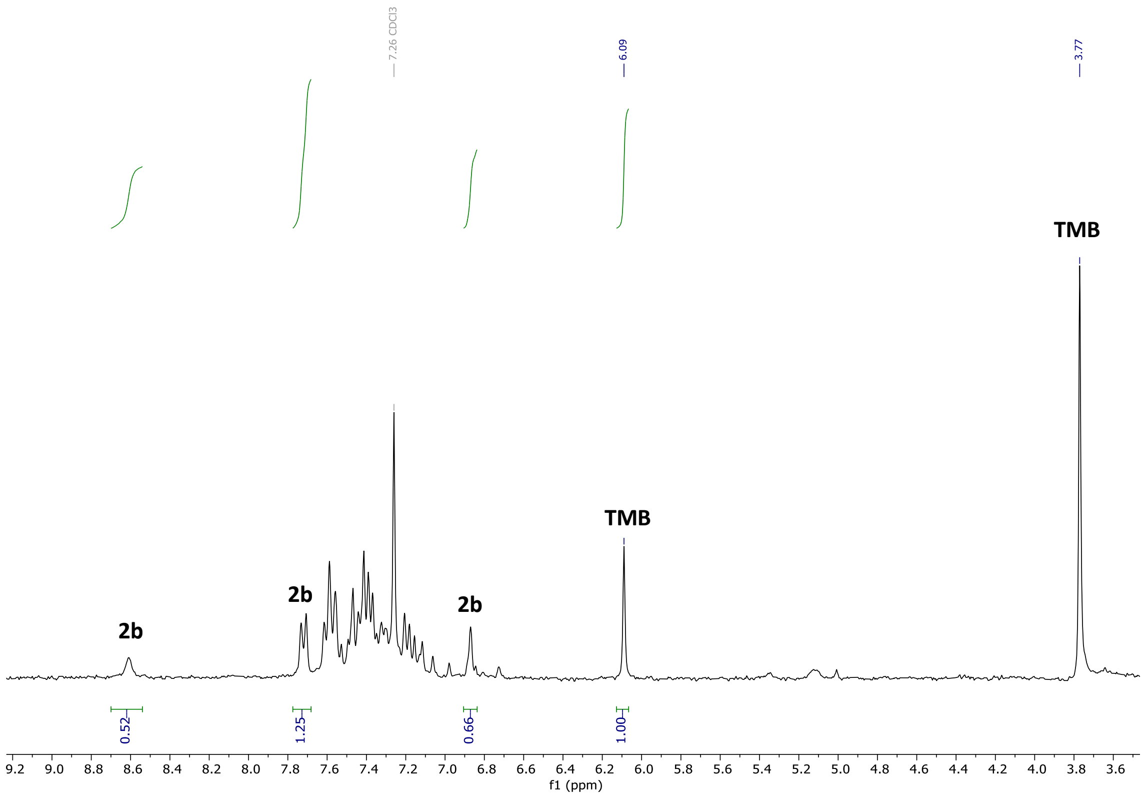

Figure S3. *^{1}H NMR of the crude of the reaction to produce **2b** in PBS/DMSO 9:1 with $Ru(bpy)_3$ and blue LEDs.*

Table S4. Control experiments in the reaction of azide **1b**.[a]

Entry	PC	Light	Yield (%)[d]
1[b]	-	-	0
2[b]	$Ru(bpy)_3$	-	0
3[b]	Eosin Y	-	0
4[c]	-	Blue	45
5[c]	$Ru(bpy)_3$	Blue	54
6[c]	-	Green	0
7[c]	Eosin Y	Green	25

*[a] Conditions: **1b** (5 μmol, 1 equiv), photocatalyst (10 mol%) if used, PBS (4.5 mL, 1 mM), DMSO (0.5 mL, 1 mM). [b] 24 h, rt. [c] blue LEDs (456 nm, 200 mW cm^{-2}, 40 W) or green LEDs (525 nm, 200 mW cm^{-2}, 40 W) situated at ≈ 4 cm from the reaction vessel, 10 min, rt. [d] Yields determined by ^{1}H NMR using 1,3,5-trimethoxybenzene as internal standard.*

S6. Spectroscopic studies

UV-Vis of substrates 1a and 1b, and products 2a and 2b

For the UV-Vis spectroscopic studies, 10 mM solutions in DMSO of substrates **1a** (or **1b**) and products **2a** (or **2b**) were freshly prepared and diluted with 1.0 mL DMSO into a quartz Hellma® fluorescence cuvette with a pathlength 10 x 4 mm, chamber volume 1.5 mL (final concentration: 10 μM). The samples were analyzed in a Jasco V-770 UV-Vis spectrophotometer at 25 °C. UV-Vis analysis were performed in the interval of 275-600 nm.

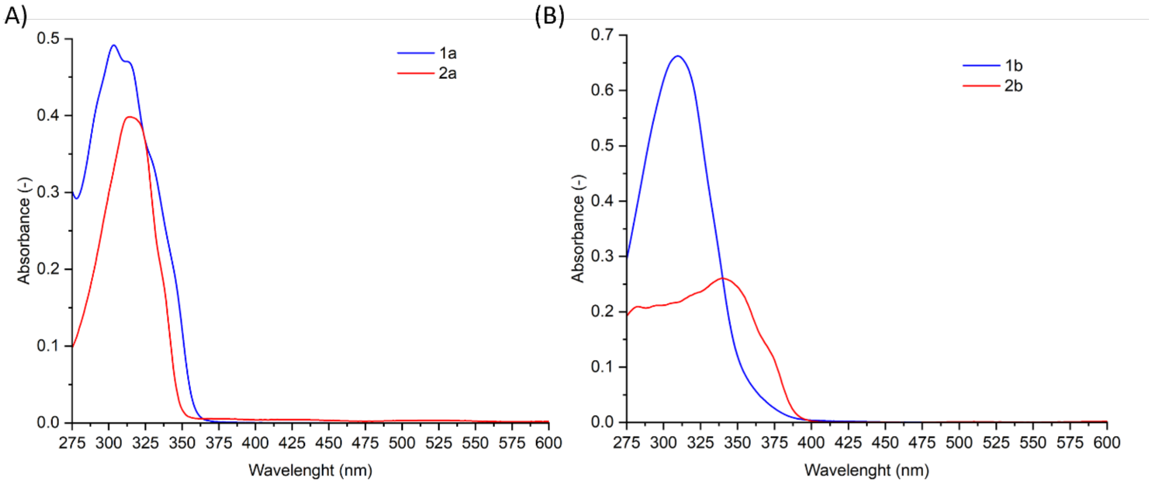

Figure S4. *UV-Vis absorption spectra of: A)* ***1a*** *(blue) and* ***2a*** *(red) (10 μM in DMSO); B)* ***1b*** *(blue) and* ***2b*** *(red) (10 μM in DMSO).*

Fluorescence spectra of substrate 1b and product 2b

For the fluorescence measurements, solutions of **1b** and **2b** (10 mM in DMSO) were freshly prepared and diluted with DMSO into quartz Hellma® fluorescence cuvette with a path length 10 x 4 mm, chamber volume 1.5 mL (final concentration: 10 μM). The samples were analyzed in a *FS5 spectrofluorometer* at 20 °C. The solutions were excited at 405 nm and the emission spectrum was recorded in the interval 425-750 nm. NOTE: we used this wavelength for the excitation because it is the one that fits with the lamp of the microscope.

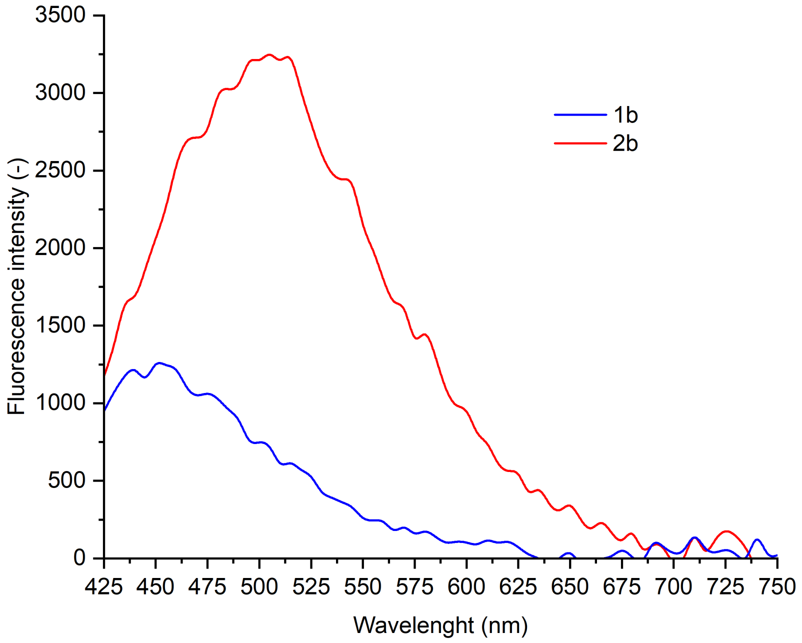

***Figure S5**. Fluorescence spectra of **1b** (blue) and **2b** (red) (10 µM in DMSO, λ_{exc} = 405 nm).*

S7. Synthesis of conjugate Eosin-CRGD

Abbreviations

DIPEA= *N,N*- Diisopropylethylamine

HBTU= *N,N,N′,N′*- Tetramethyl-O-(1*H*-benzotriazol-1-yl)uranium hexafluorophosphate

DMF= Dimethylformamide

TFA= Trifluoroacetic acid

TCEP= Tris(2-carboxyethyl)phosphine

TIPS= Triisopropylsilane

Synthesis of tetrapeptide CRGD[4]

Peptide **CRGD**: (C= Cysteine, R= Arginine, G= Glycine and D= Aspartic Acid) was synthesized on a 0.1 mmol scale using the Fmoc-based amino acid protection strategy on H-Rink amide *ChemMatrix®* resin using a HBTU as activating agent, DIPEA as base, and DMF as solvent. The removal of the temporary Fmoc protecting group was performed by treating the resin with 20% of piperidine in DMF for 20 min in two times. Finally, the resin was washed twice with DMF.

The cleavage/deprotection step was performed by treating the resin-bound peptide for 2 h with the following cleavage cocktail: 3.6 mL of trifluoroacetic acid (TFA), 200 µL of dichloromethane, 100 µL of H_2O and 100 µL of triisopropylsilane (TIPS) (4 mL of cocktail/ 200 mg resin). The resin was filtered, and TFA filtrate was concentrated with a nitrogen current to an approximate volume of 3.0 mL, which was added to ice-cold diethyl ether (45.0 mL). After 10 min, the precipitate was centrifugated and washed again with 45.0 mL of ice-cold ether. The solid residue was dried under nitrogen, dissolved in water and purified by preparative reverse-phase HPLC, 20.0 mL/min, gradient 0 to 40% B over 22 min (A: H_2O 0.1% TFA, B: MeCN 0.1% TFA) on a preparative *Agilent 1260 Infinity II* using a Luna 5u C18(2) 100A (250 x 10 mm, 5 µm) reverse-phase column from Phenomenex.

The tetrapeptide was analyzed by analytical HPLC-MS on a *THERMO Ultimate 3000* coupled to a *Bruker AmaZon SL ion trap LC-MS* using a flow rate of 0.35 mL/min at room temperature. The initial conditions for the solvent system were H_2O/MeCN (95:5) followed by a gradual change over 12 min to H_2O/MeCN (5:95).

ESI/MS (m/z): Calculated for $C_{15}H_{28}N_8O_6S$ [M] = 448.19; found: 448.34 [M]

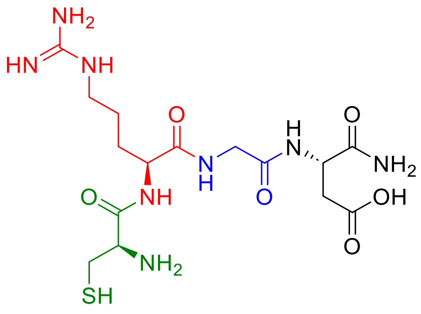

CRGD

Figure S6*. Representation peptide CRGD (C= Cysteine, R= Arginine, G= Glycine and D= Aspartic Acid).*

Synthesis of conjugate Eosin-CRGD

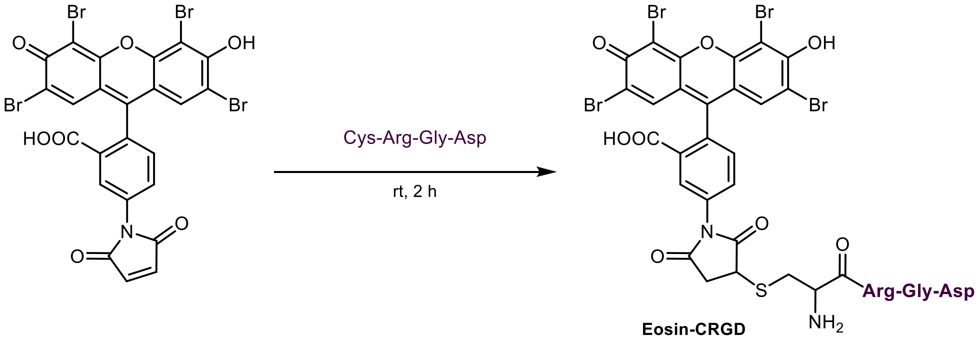

The tetrapeptide **CRGD** was dissolved in degassed TrisHCl at pH 7-7.5 for a final concentration of 50 μM (13.4 mL), and excess of tris-carboxyethylphosphine (TCEP) was added to the solution (ratio TCEP/peptide 10:1, 1.9 mg) to ensure that disulfide bonds are reduced. The solution was stirred at room temperature for 30 minutes and were mixed with 670 μL solution of 5-maleimido-Eosin (commercially available) in DMSO (10 mM, ratio Eosin/peptide 10:1). After 2 h at rt, the crude, that showed several peaks, was purified by preparative reverse-phase HPLC, 20.0 mL/min, gradient 0 to 95% B over 23 min (A: H_2O 0.1% TFA, B: MeCN 0.1% TFA) on a preparative *Agilent 1260 Infinity II* using a Luna 5u C18(2) 100A (250 x 10 mm, 5 mm) reverse-phase column from Phenomenex.

The resulting peptide was characterized by analytical HPLC-MS on a *THERMO Ultimate 3000* coupled to a *Bruker AmaZon SL ion trap LC-MS* using a flow rate of 0.35 mL/min at room temperature. The initial conditions for the solvent system were H_2O/MeCN (95:5) followed by a gradual change over 12 min to H_2O/MeCN (5:95).

Yield: 75% (0.001 mmol, 0.6 mg)

ESI/MS (m/z): Calculated for $C_{39}H_{37}Br_4N_9O_{13}S$ [M] = 1191.45; found: 1192.17 $[M]^+$

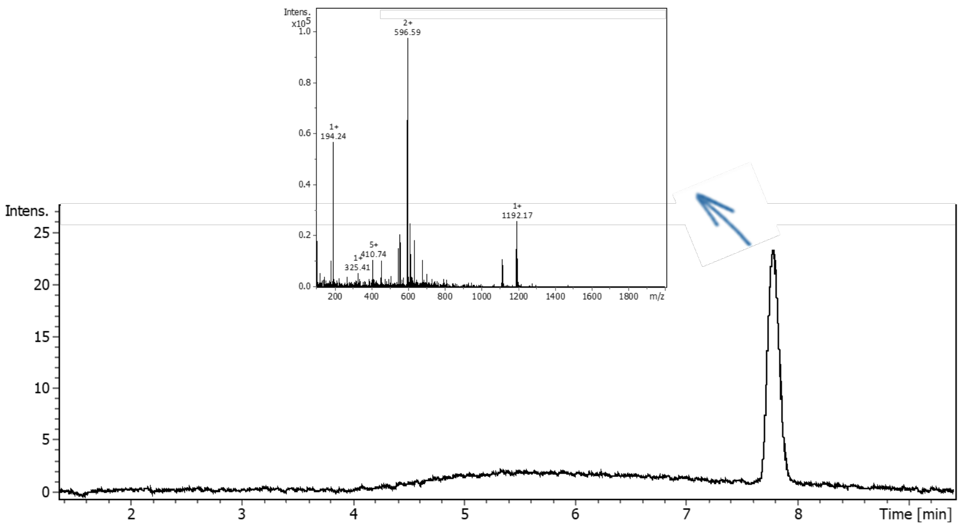

Figure S7. *LC-MS analysis of* ***Eosin-CRGD****, with UV detection at 544 nm, and the corresponding mass spectrum*

S8. General information for the cellular experiments

All steps were performed on a sterile clean bench *Tesltar AV-100* at room temperature. Solutions stored in a fridge were warmed beforehand in a water bath (37 °C). Unless otherwise specified, all incubations were performed in DMEM.

Cell Cultures: All cell lines were cultured in DMEM (Dulbecco´s modified Eagle´s medium), 5 mM glutamine, penicillin (100 units/mL) and streptomycin (100 units/mL) (all from *Invitrogen*). Proliferating cultures were maintained in a 5% CO_2 humidified incubator at 37 °C.

For all the experiments, cells were seeded in well plates at the indicated concentration two days before treatment.

Protein quantification: For protein concentration measurements the Bio-Rad *DC* Protein Assay Kit was used (*Bio-Rad* 500-0114).

Fluorescence microscopy: All images were obtained with an *Andor Zyla* mounted on a *Nikon TiE*. Confocal images were acquired in an *Andor Dragonfly* High Speed Confocal Platform. Images were further processed with *Image J* or *NIS* software (*Nikon*).

Microscopy settings: The filter sets for the observation of the fluorescence of the products were as follows:

Widefield: LED λ excitation: 405 nm. Filter cube DAPI-1160B-000 (*Semrock*): BP 387/11-25 nm, LP 447/60-25 nm and DM 409 nm. Confocal: Laser excitation: 405 nm. LP 450/50 and DM 418 nm.

S9. Cell viability

The toxicity was measured using different assays in HeLa cell lines. For the studies, 125000 cells per well were seeded in 96 well plates 2 days before performing the assay.

Cell Counting Kit-8 (CCK-8): Cells were incubated with different concentrations of either $Ru(bpy)_3(PF_6)_2$, Eosin Y, substrates **1a**,**b** and products **2a**,**b** in DMEM. After 15 min of incubation, cells were washed twice with PBS and then irradiated in DMEM-HEPES with blue or green LEDs for 15-45 minutes following the indicated set-up. Then, 10 µL of the CCK-8 solution was carefully added to each well of the plate to avoid the introduction of bubbles. Cells were then incubated for 3 h to allow the formation of formazan precipitates by metabolically active cells. The quantity of formazan in each well (directly proportional to the number of viable cells) was measured by recording changes in absorbance at 450 nm in a microtiter plate reading spectrophotometer *(Tecan Infinite 200 PRO*).

Representative CCK-8 studies:

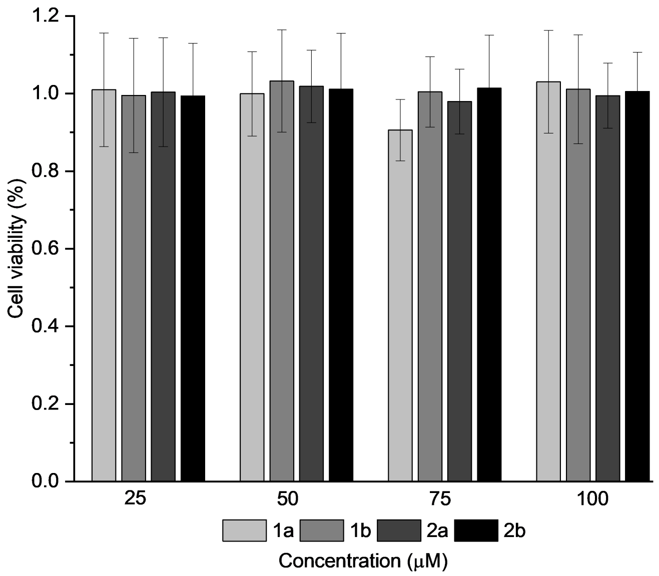

***Figure S8**. Viability studies with substrates and products in absence of irradiation. Bars representation of the viability of cells treated with either substrates **1a, 1b** or products **2a, 2b** (25-100 µM) for 15 min and then washed twice with PBS and incubated in HEPES-DMEM for 15 min. The viability is expressed as the fold change of the absorbance value with respect to untreated cells (value 1.0). The error bars represent the standard deviation of three different samples.*

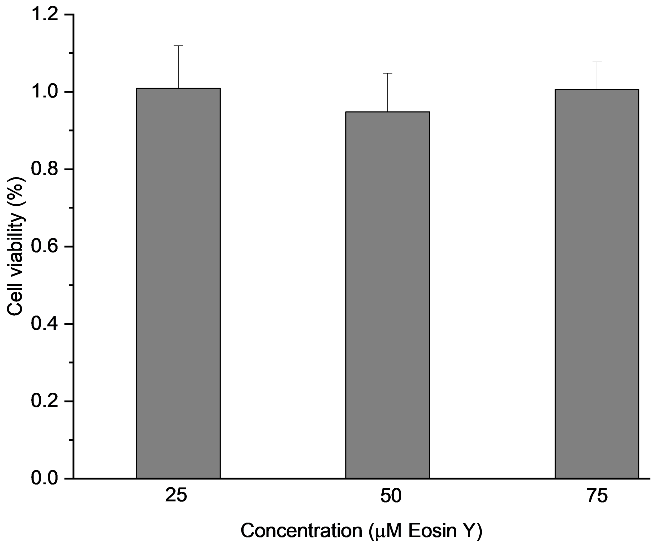

Figure S9. *Viability studies in absence of irradiation. Bars representation of the viability of cells treated with Eosin Y (25-75 μM) for 15 min and then washed twice with PBS and incubated in HEPES-DMEM for another 15 min. The viability is expressed as the fold change of the absorbance value with respect to untreated cells (value 1.0). The error bars represent the standard deviation of three different samples.*

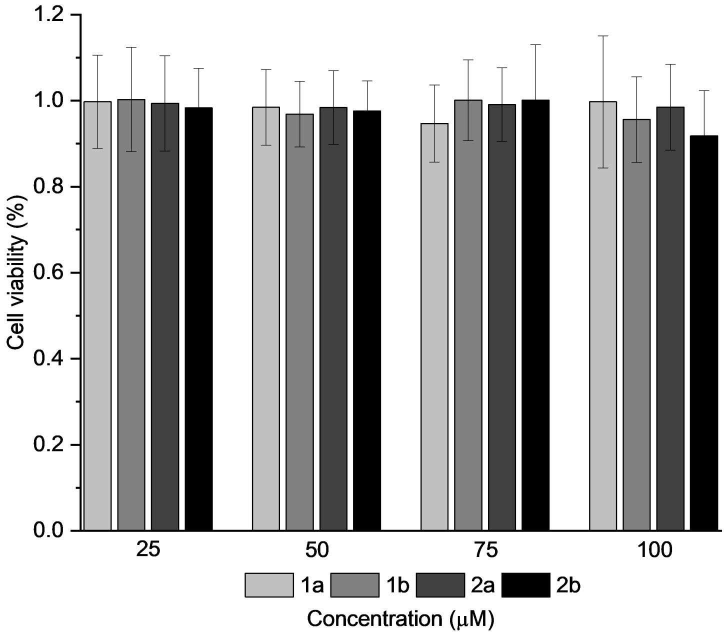

Figure S10. *Viability studies with substrates and products under green LED irradiation. Bars representation of the viability of cells pretreated with either substrates **1a, 1b** or products **2a, 2b** (25-100 μM) for 15 min and then washed twice with PBS. Cells were then irradiated in HEPES-DMEM under green LEDs for another 15 min. The viability is expressed as the fold change of the absorbance value with respect to untreated cells (value 1.0). The error bars represent the standard deviation of three different samples.*

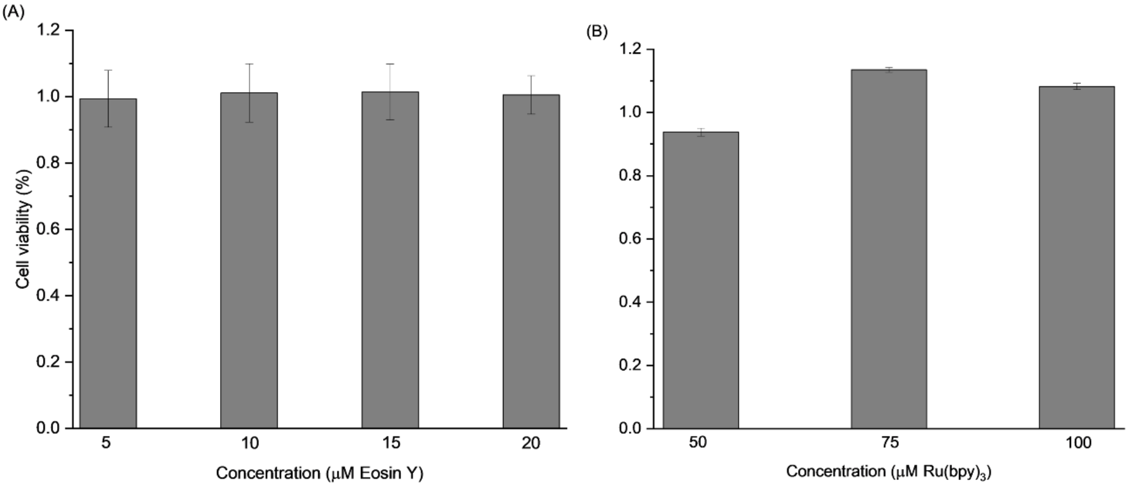

***Figure S11**. Viability studies with photocatalysts under blue or green irradiation. Bars representation of the viability of cells treated with the photocatalyst for 15 min and then washed twice with PBS. Cells were then irradiated in HEPES-DMEM under light irradiation for another 15 min. A) Eosin Y (5-20 μM) and green LEDs; B) $Ru(bpy)_3$ (50-100 μM) and blue LEDs. The viability is expressed as the fold change of the absorbance value with respect to untreated cells (value 1.0). The error bars represent the standard deviation of three different samples.*

It is important to note that under these irradiation conditions the cells were mostly alive at the end of the experiment (90% of cell survival). As expected, higher concentrations of Eosin Y led to more product **2a**, but at the expense of compromising the viability of the cells (>50% cell death at 50 μM).

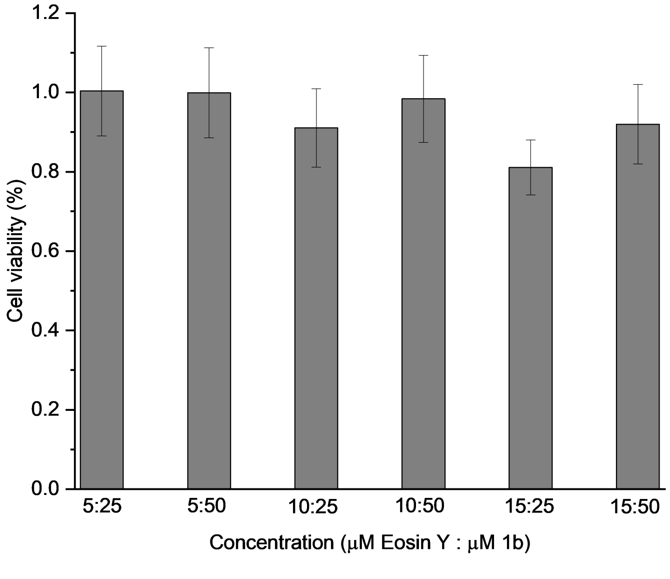

***Figure S12**. Viability studies of cells after the photocatalyzed reactions. Bars representation of the viability of cells pretreated with substrate **1b** (25-50 μM) and Eosin Y (5-15 μM) for 15 min and then washed twice with PBS. Cells were then irradiated in HEPES-DMEM under green LEDs for another 15 min. The viability is expressed as the fold change of the absorbance value with respect to untreated cells (value 1.0). The error bars represent the standard deviation of three different samples.*

S10. Photocatalytic reactions in cell cultures

The experiments were performed with 3-5 million cells per plate (100 mm) seeded two days before treatment. Just before the experiments fresh DMEM was added to cells that had been seeded on glass plates. Then, they were mixed with the photocatalyst and the azide **1a** or **1b** (50 µM, final volume of 3 mL) for 15 min, using freshly prepared stock solution (10 mM in DMSO). Cells were washed twice with PBS and then with the medium replaced by HEPES-DMEM (3 mL) and the plates irradiated for the indicated reaction time with the appropriate irradiation source.

As a representation, in illustration S2 is shown the set-up for 100 mm well plates irradiated at 15 cm of distance under fan refrigeration.

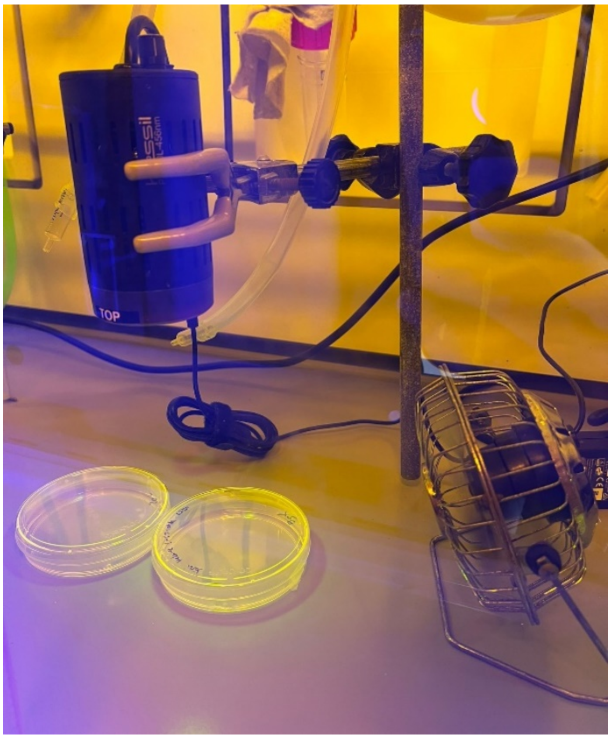

Illustration S2. *Set-up for the irradiation of cells.*

S11. Quantification studies of experiments with cells using LC/MS

Cellular reactions with azide 1a

Calibration curve of indole product (2a)

Stocks of this product at different concentrations (1 - 60 µM) plus an internal standard at constant concentration (coumarin, 20 µM) in CH_3CN were prepared. For the calibration curve,

we represented the (product/internal standard) peak area ratio obtained in the UV 310 nm HPLC spectra vs the concentration.

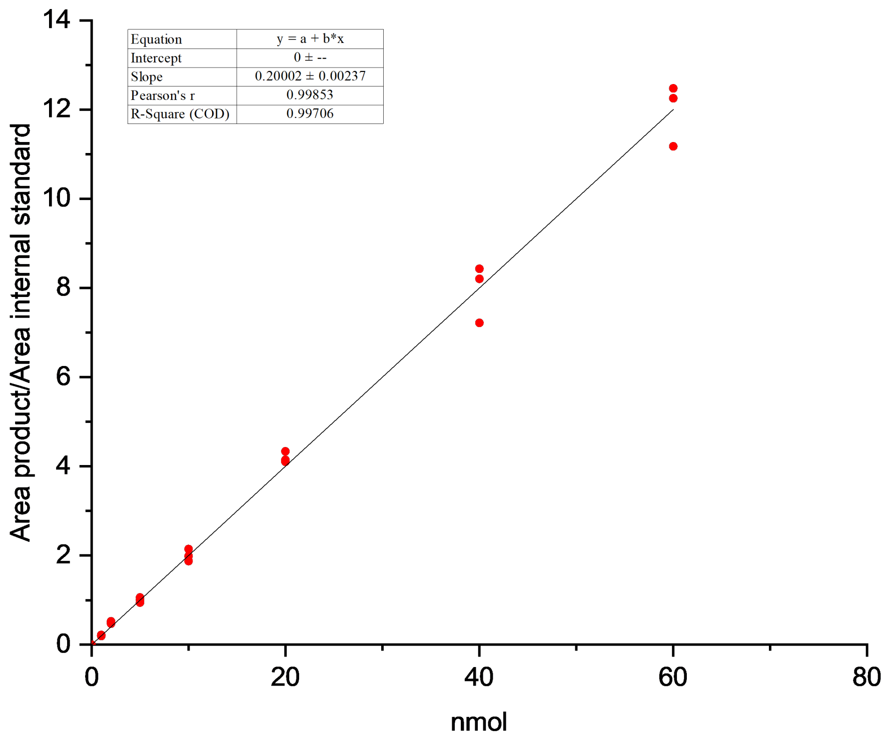

Figure S13. *Calibration curve of product* ***2a***.

Cellular experiments with 1a

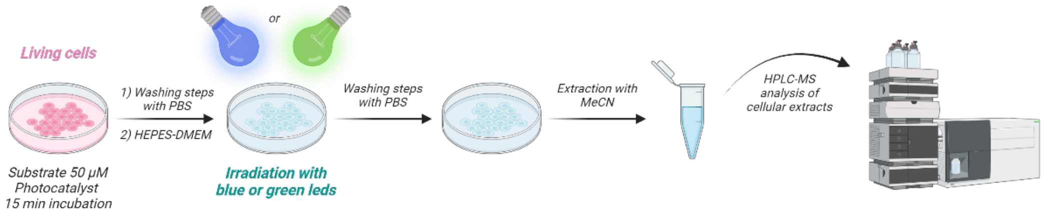

Figure S14. *Schematic representation for the photocatalytic reactions in living cells and quantification of the product*

The protocol required previous optimization of the concentration of substrates and photocatalysts, incubation time, irradiation time, light source and distance of irradiation.

HeLa Cells were mixed with catalyst $Ru(bpy)_3(PF_6)_2$ (at different concentration from 25 µM to 75 µM) or Eosin Y (from 5 to 20 µM), and azide **1a** (50 µM) in DMEM (final volume of 3 mL) for 15 min. Prior to irradiation, the cells were washed twice with PBS and then placed HEPES-DMEM. Plates containing cells were disposed as shown in illustration S2, and were irradiated with a

Kessil PR160 at 15 cm of distance (approx. 20 mW cm^{-2}) under fan refrigeration for the indicated time (45 min with the ruthenium PC or 15 min when using Eosin Y).

The measurements were made in individual plates. Therefore, for each plate the reaction media was collected in a 15 mL falcon. Then cells were washed with 3 mL of PBS (x2) and were collected separately in two 15 mL Falcons. Then, the cell monolayer was treated three times with 1 mL of CH_3CN for extracting cellular compounds. After 5 min of pipetting up and down, this solution was transferred to a 15 mL Falcon. Finally, 3 mL of acetonitrile extracts were obtained. The washings and cellular extracts were lyophilized and analyzed.

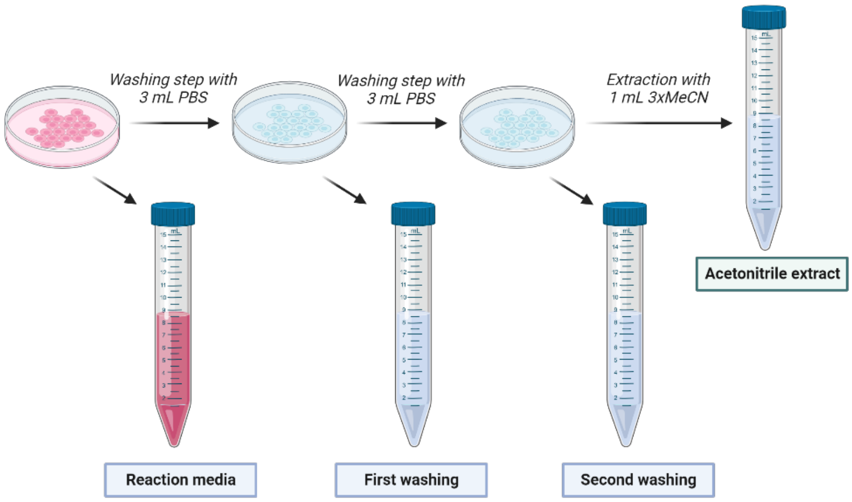

Figure S15. *Schematic representation of the protocol for the cellular treatment after irradiation.*

Quantification of indole product 2a formed in the cellular experiments

For the analysis, the final samples (reaction media, washings, and acetonitrile extracts) were dissolved in 1 mL of CH_3CN and filtered through Whatman® syringe filters of 0.45 μM pore size. Then, 196 μL were transferred to an Eppendorf, and coumarin (internal standard, stock of 1 mM in CH_3CN) was added to reach a final concentration of 20 μM. The samples were injected in an *Agilent 1260 Infinity II* coupled to an *Agilent Technologies 6120 Quadrupole LC-MS* using a Phenomenex *Luna-C$_{18}$* (250 x 10 mm) reverse-phase column and a flow rate of 0.35 mL/min at room temperature. For the solvent system, initial conditions 5% B (A: H_2O 0.1% TFA, B: CH_3CN 0.1% TFA) were used followed by a gradual change over 12 min to 95% of B. Then, initial conditions were recovered in a gradual change over 1 min, and maintained for 2 min. The chromatogram was recorded using UV detection at λ = 310 nm.

Representative results obtained after 45 min of reaction using Ru(bpy)$_3$(PF$_6$)$_2$ as photocatalyst

The samples of each well were analyzed and the amount of product **2a** (nmol) formed was calculated using the calibration curve. As a representative example, we show here the experiments with catalyst Ru(bpy)$_3$(PF$_6$)$_2$ (50 and 75 µM) and azide **1a** (50 µM) irradiated with a Blue Kessil PR160 at 15 cm of distance under fan refrigeration for 45 min.

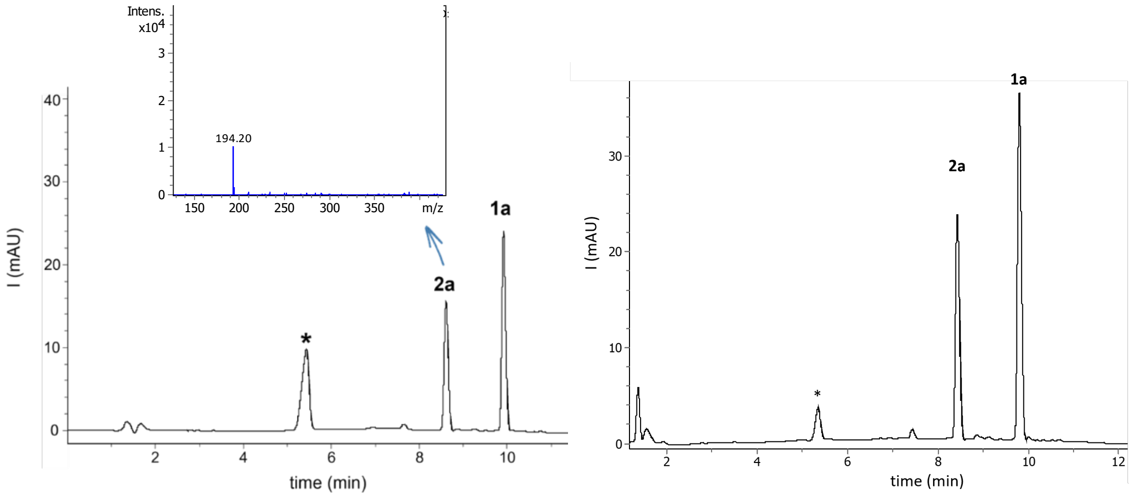

***Figure S16**. Detection of **2a** in HeLa cells (acetonitrile extract), chromatogram with UV detection (310 nm, coumarin used as internal standard*, rt: 5 min) and inset with the mass spectrum corresponding 25to the peak of **2a**. Results when using 50 µM of Ru(bpy)$_3$(PF$_6$)$_2$ (left),* and *75 µM (right).*

Table S5. Quantification of indole **2a** in the acetonitrile extracts of **HeLa cells** after the experiments with Ru(bpy)$_3$(PF$_6$)$_2$ (**50 µM**) and azide **1a** (50 µM), irradiated for 45 min. Three experiments performed in different plates, with cells coming from the same culture.

**Sample**	**Number of cells**	**Value (nmol)**	**Normalization (nmol / 10^6 cells)**
1	3.9 *10^6	3.33	0.86
2	3.9*10^6	5.25	1.32
3	3.9*10^6	4.35	1.10
**Average**		4.26 ± 0.90	1.09 ± 0.23

Table S6. Quantification of the indole **2a** formed in **HeLa cells** without irradiation and without photocatalyst, under ambient light, in an experiment performed in one plate.

**Sample**	**Normalization (nmol / 10^6 cells)**
Reaction media	0.03

First washing	0.03
Second washing	0.00
Acetonitrile extraction	0.16
**Total value**	0.19

Table S7. Quantification of indole **2a** in **HeLa cells**, after the experiments with azide **1a** (50 µM), irradiated with blue light for 45 min, in absence of the photocatalyst. Three experiments performed in different plates, with cells coming from the same culture.

Sample	Number of cells	Value (nmol)	Normalization (nmol / 10^6 cells)
1	$5.2*10^6$	2.17	0.41
2	$5.2*10^6$	3.26	0.62
3	$5.2*10^6$	1.46	0.28
**Average**		2.30 ± 0.91	0.35 ± 0.17

Table S8. Quantification of indole **2a** in **HeLa cells**, after the experiments with $Ru(bpy)_3(PF_6)_2$ (**75 µM**) and azide **1a** (50 µM), irradiated for 45 min. Three experiments performed in different plates, with different cell cultures.

Sample	Number of cells	Value (nmol)	Normalization (nmol / 10^6 cells)
1	$2.9*10^6$	4.21	1.45
2	$3.9*10^6$	5.40	1.37
3	$4.4*10^6$	9.72	2.24
**Average**		6.44 ± 2.90	1.69 ± 0.48

Table S9. Example of the comparison of the average product **2a** detected in **HeLa cells**, in the media and the washings, in an experiment performed in one plate using $Ru(bpy)_3(PF_6)_2$ (**75 µM**) and azide **1a** (50 µM), irradiated for 45 min.

Sample	Normalization (nmol / 10^6 cells)
Reaction media	0.09
First washing	0.08
Second washing	0.06
Acetonitrile extraction	1.41
**Total value**	1.64

We have also performed experiments using cells from different cultures and the results were compared considering the number of cells per plate in each experiment. After the normalization, comparable data were obtained, confirming the reproducibility of the reaction.

Importantly, the amount of product in the cellular extracts depends on the number of cells so far used, further confirming the intracellular character of the process.

Table S10. Quantification of indole **2a** in **HeLa cells**, after the experiments performed in two different days, with different cell cultures, using $Ru(bpy)_3(PF_6)_2$ (**50 µM**) and azide **1a** (50 µM), irradiated for 45 min.

**Sample A**	**Number of cells**	**Value (nmol)**	**Normalization (nmol / 10^6 cells)**
1	$3.9*10^6$	3.33	0.86
2	$3.9*10^6$	5.25	1.32
3	$3.9*10^6$	4.35	1.10
**Average**		4.26 ± 0.90	1.09 ± 0.23

**Sample B**	**Number of cells**	**Value (nmol)**	**Normalization (nmol / 10^6 cells)**
1	$2.9*10^6$	2.72	0.94
2	$2.9*10^6$	3.16	1.09
3	$2.9*10^6$	3.38	1.17
**Average**		3.09 ± 0.34	1.06 ± 0.12

Table S11. Quantification of indole **2a** in **A549 cells**, after the experiments with $Ru(bpy)_3(PF_6)_2$ (**75 µM**) and azide **1a** (50 µM), irradiated for 45 min. Three experiments performed in different plates, with cells coming from the same culture.

**Sample**	**Number of cells**	**Value (nmol)**	**Normalization (nmol / 10^6 cells)**
1	$4*10^6$	5.28	1.32
2	$4*10^6$	4.04	1.01
3	$4*10^6$	7.93	1.98
**Average**		5.75 ± 1.99	1.44 ± 0.03

Table S12. Quantification of indole **2a** in **HeLa cells**, after the experiments performed with Eosin Y (**10 μM**) as photocatalyst and azide **1a** (50 μM), irradiated with green light for 15 min. Three experiments performed in different plates, with cells coming from the same culture.

Sample	Number of cells	Value (nmol)	Normalization (nmol/ 10^6 cells)
1	$4.2*10^6$	0.83	0.20
2	$4.2*10^6$	0.94	0.22
3	$4.2*10^6$	1.15	0.27
**Average**		0.97 ± 0.16	0.23 ± 0.04

Quantification of the intracellular content of $Ru(bpy)_3(PF_6)_2$

Calibration curve of $Ru(bpy)_3(PF_6)_2$

Stocks of the $Ru(bpy)_3(PF_6)_2$ at different concentrations (1 - 250 μM) plus an internal standard at constant concentration (caffeine, 20 μM) in CH_3CN/Water (50/50) were prepared. For the calibration curve, we represented the (photocatalyst/internal standard) peak area ratio obtained in the MS through *Extracted Ion Chromatogram* HPLC spectra vs the concentration.

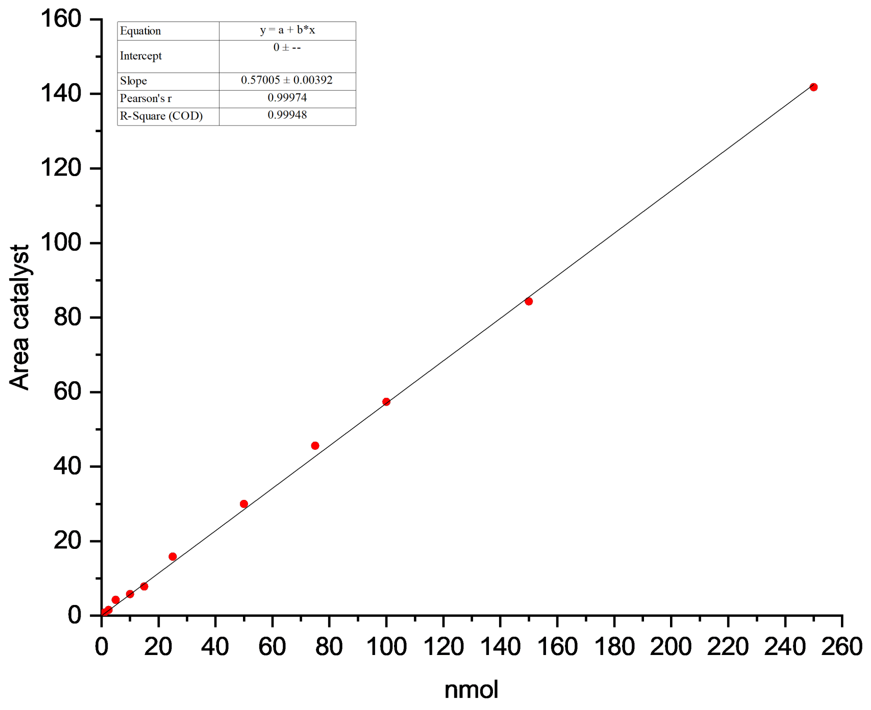

Figure S17 *Calibration curve of catalyst Ru(bpy)₃.*

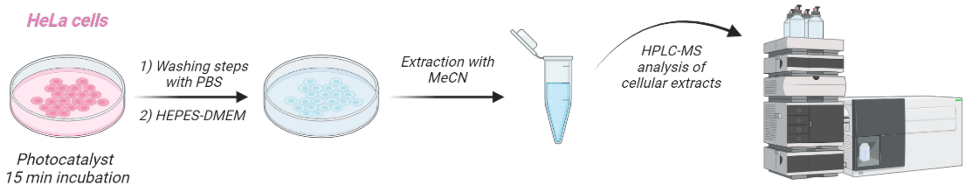

Figure S18. *Schematic representation for the quantification of photocatalysts*

HeLa Cells were mixed with catalyst **Ru(bpy)$_3$** (at different concentration from 25 µM to 75 µM) in DMEM (final volume of 3 mL) for 15 min. After the incubation, the cells were washed twice with PBS and then mixed with fresh HEPES-DMEM (3 mL). For each plate the reaction media was collected in a 15 mL falcon. Then, the cell monolayer was treated with 1 mL of CH_3CN for extracting cellular compounds. After 5 min of pipetting up and down, this solution was transferred to an Eppendorf.

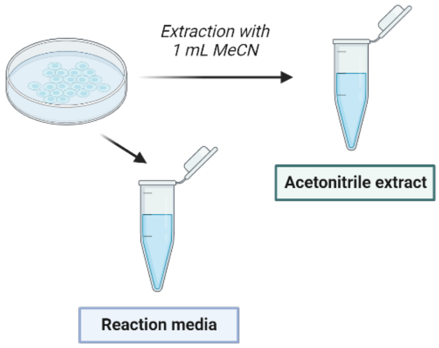

Figure S19. *Schematic representation of the protocol for the cellular extraction.*

The washings and cellular extracts were filtered through a Whatman® syringe filters of 0.45 µM pore size and analyzed. Then, 460 µL were transferred to an Eppendorf, and 40 µL of caffeine (internal standard, stock of 500 µM in CH_3CN) were added to reach a final concentration of 20 µM. Finally, 500 µL of Milli-Q water was introduced in the Eppendorf.

The samples were injected in a *THERMO Ultimate 3000* coupled to a *Bruker AmaZon SL ion trap LC-MS* using a column *XBridge Premier BEH Phenyl Column C_{18}* (2.1 mm X 150 mm) and a flow rate of 0.35 mL/min at room temperature. For the solvent system, initial conditions 10% B (A: H_2O 0.1% Formic acid, B: CH_3CN 0.1% Formic acid) were used followed by a gradual change over 23 min to 75% of B. Then, initial conditions were recovered in a gradual change over 1 min, and

maintained for 2 min. The chromatogram was recorded *via* MS through *Extracted Ion Chromatogram* HPLC spectra.

The amount of $Ru(bpy)_3$ (nmol) was calculated using the calibration curve, which revealed the absence of this compound in the HEPES medium.

Representative results obtained after 15 min of incubation with $Ru(bpy)_3$ (50 μM)

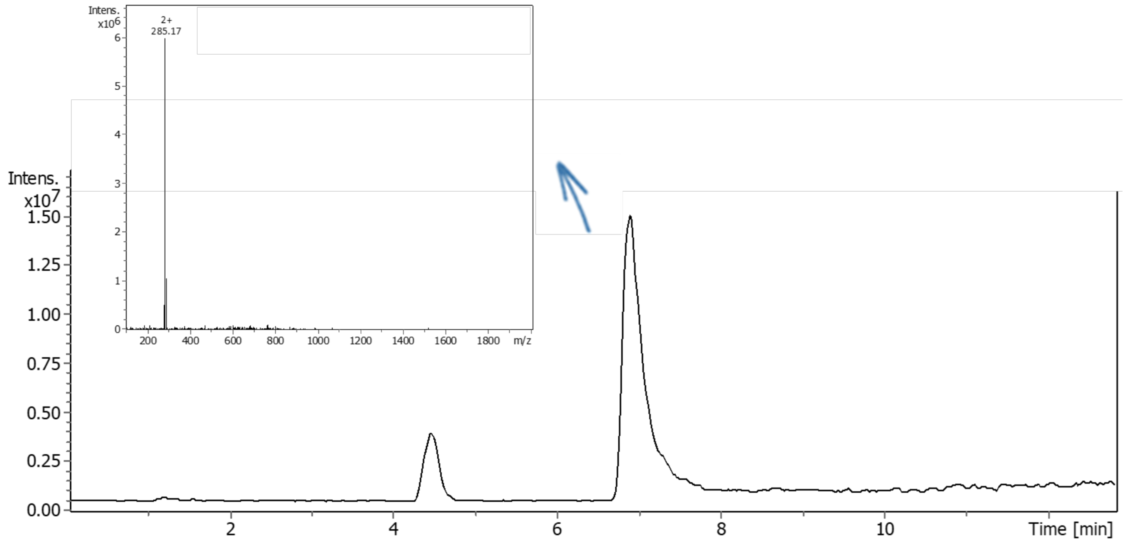

Figure S20. *Representative detection of* ***$Ru(bpy)_3$*** *in HeLa cells (acetonitrile extract), chromatogram with MS detection (caffeine used as internal standard, rt: 4.5 min) and inset with the mass spectrum corresponding to the peak. Results when using 50 μM of $Ru(bpy)_3$.*

Table S13. Quantification of **$Ru(bpy)_3$** inside **HeLa cells**, in the experiments using **50 μM**, after incubation of 15 min. Three experiments performed in different plates with cells proceeding from the same culture.

Sample	Number of cells	Value (nmol)	Normalization (nmol / 10^6 cells)
1	$4*10^6$	0.23	0.06
2	$4*10^6$	0.25	0.06
3	$4*10^6$	0.28	0.07
**Average**		0.26 ± 0.03	0.06 ± 0.006

Table S14. Quantification of **$Ru(bpy)_3$** (**50 μM**) inside **HeLa cells** in an experiment performed in one plate.

Sample	Value (nmol)

Reaction media (DMEM-HEPES)	0.00
Acetonitrile extraction	0.25
**Total value**	0.25

Cellular reactions with azide 1b

Calibration curve of indole 2b

Stocks of product at different concentrations (0.25 - 50 μM) plus an internal standard at constant concentration (coumarin, 20 μM) in CH_3CN were prepared. For the calibration curve, we represented the (product/internal standard) peak area ratio obtained in the UV 310 nm HPLC spectra vs the concentration.

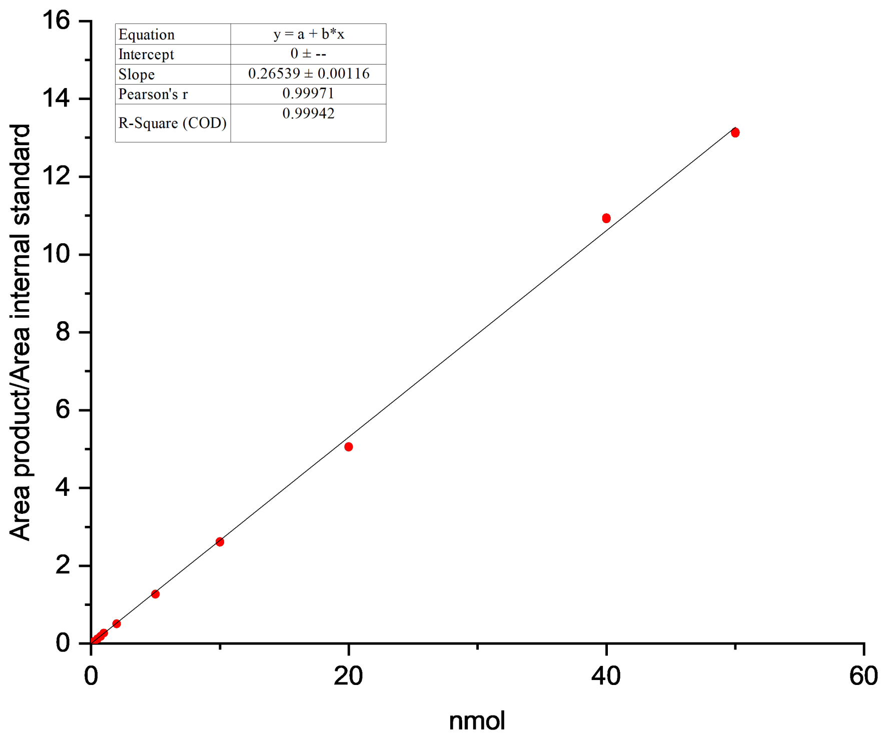

Figure S21. *Calibration curve of product* ***2b***.

Experiments in cells with 1b

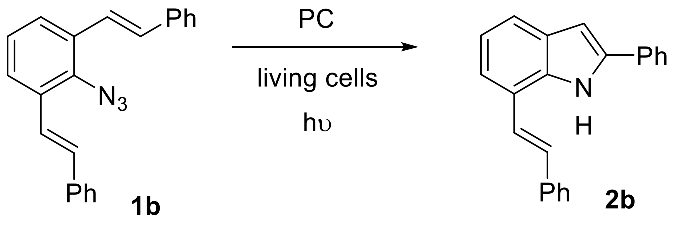

The experiments were carried out using the same protocol than with **1a**. The samples after the experiments with cells (reaction media, washings and acetonitrile extract) were dissolved in 1 mL of CH_3CN and filtered through a Whatman® syringe filters of 0.45 μM pore size. Then, 196 μL were transferred to an Eppendorf, and coumarin (internal standard, stock of 1 mM in CH_3CN) was added to reach a final concentration of 20 μM. The samples were injected in a *THERMO Ultimate 3000* coupled to a *Bruker AmaZon SL ion trap LC-MS* using a column *XBridge Premier BEH Phenyl Column* C_{18} (2.1 mm X 150 mm) and a flow rate of 0.35 mL/min at room temperature. For the solvent system, initial conditions 20% B (A: H_2O 0.1% Formic acid, B: CH_3CN 0.1% Formic acid) were used followed by a step at 5 min to 70% B, at 9 min to 95% B, and a gradual change over 11 min to 20% of B. Then, initial conditions were recovered in a gradual change over 1 min, and maintained for 2 min. The chromatogram was recorded *via* UV absorption at λ = 300 nm.

Quantification of indole product 2b formed in the cellular experiments

The samples of each well were analyzed and the amount of product **2b** (nmol) formed was calculated using the calibration curve. As a representative example, we show here the experiment with catalyst Eosin Y (10 µM) and azide **1b** (50 µM) irradiated with a Green Kessil PR160 at 15 cm of distance under fan refrigeration for 15 min in HeLa cells (525 nm, 40 W, 20 mW cm^{-2}).

Representative results obtained after 15 min of reaction using Eosin Y as photocatalyst

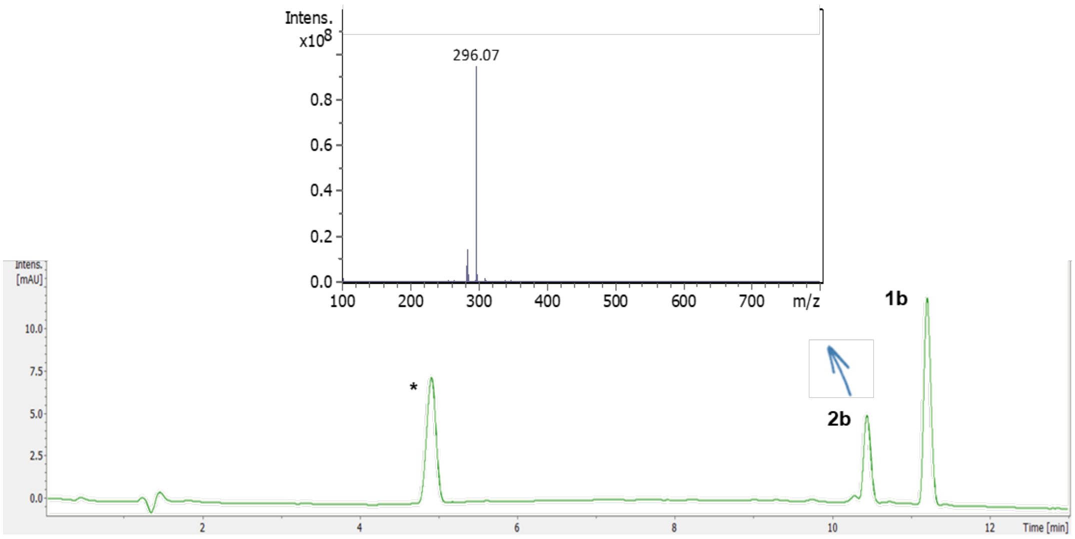

Figure S22. *Representation detection of* ***2b*** *in HeLa cells (acetonitrile extract), chromatogram with UV detection (300-330 nm, coumarin used as internal standard, rt: 5 min) and inset with the mass spectrum corresponding to the peak of* ***2b***. *Results when using 20 µM of Eosin Y.*

Table S15. Quantification of the indole **2b** obtained **in HeLa cells**, after the experiments using Eosin Y (**20 µM**) as photocatalyst and azide **1b** (50 µM), irradiated for 15 min. Three experiments performed in different plates with cells proceeding from the same culture.

Sample	Number of cells	Value (nmol)	Normalization (nmol / 10^6 cells)
1	$3*10^6$	1.37	0.46
2	$3*10^6$	1.76	0.59
3	$3*10^6$	1.19	0.40
**Average**		1.44 ± 0.29	0.48 ± 0.10

Table S16. Quantification of indole **2b** in **HeLa cells**, after the experiments with azide **1b** (50 µM), irradiated for 15 min, in absence of the photocatalyst. Three experiments performed in different plates, with cells coming from the same culture.

Sample	Number of cells	Value (nmol)	Normalization (nmol / 10^6 cells)
1	$4.6*10^6$	0.39	0.09
2	$4.6*10^6$	0.37	0.08
3	$4.6*10^6$	0.42	0.09
**Average**		0.39 ± 0.08	0.08 ± 0.005

Table S17. Quantification of indole **2a** in **HeLa cells** in experiments performed with cells obtained from different cultures, using Eosin Y (**20 µM**) as photocatalyst and azide **1b** (50 µM), 15 min irradiation.

Sample 1	Number of cells	Value (nmol)	Normalization (nmol / 10^6 cells)
1	$3*10^6$	1.37	0.46
2	$3*10^6$	1.76	0.59
3	$3*10^6$	1.19	0.40
**Average**		1.44 ± 0.29	0.48 ± 0.10

Sample 2	Number of cells	Value (nmol)	Normalization (nmol / 10^6 cells)
1	$4.3*10^6$	1.88	0.43
2	$4.3*10^6$	1.64	0.38
3	$4.3*10^6$	1.6	0.37

**Average**	1.71 ± 0.15	0.39 ± 0.03

Quantification of photocatalyst Eosin Y after incubation with HeLa cells

Calibration curve of Eosin Y

Stocks of Eosin Y at different concentrations (0.05 - 10 μM) in CH_3CN were prepared. For the calibration curve, we represented the photocatalyst peak area ratio obtained in the MS through *Extracted Ion Chromatogram* HPLC spectra vs the concentration.

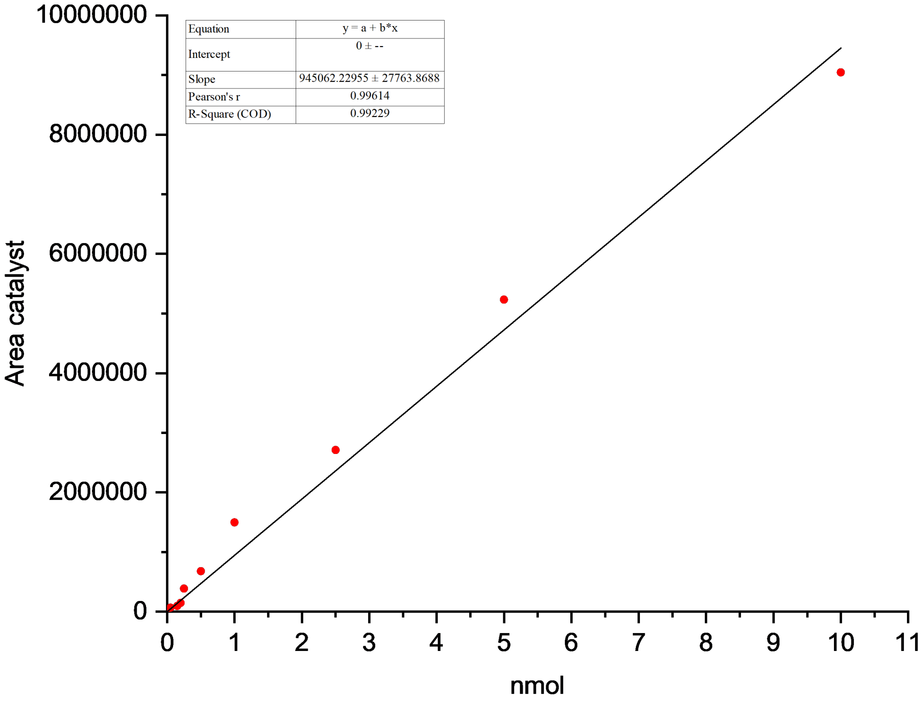

Figure S23. *Calibration curve of catalyst Eosin Y.*

The cellular quantification was carried out using the same protocol than for **$Ru(bpy)_3$**. The samples after the experiments with cells (reaction media and acetonitrile extract) were filtered through a Whatman® syringe filters of 0.45 μM pore size.

The samples were injected in a *THERMO Ultimate 3000* coupled to a *Bruker AmaZon SL ion trap LC-MS* using a column *XBridge Premier BEH Phenyl Column C_{18}* (2.1 mm X 150 mm) and a flow rate of 0.35 mL/min at room temperature. For the solvent system, initial conditions 20% B (A: H_2O 0.1% Formic acid, B: CH_3CN 0.1% Formic acid) were used followed by a step at 5 min to 70% B, at 9 min to 95% B, and a gradual change over 11 min to 20% of B. Then, initial conditions were

recovered in a gradual change over 1 min, and maintained for 2 min. The chromatogram was recorded *via* MS through *Extracted Ion Chromatogram* HPLC spectra.

The amount of **Eosin Y** (nmol) was calculated using the calibration curve. As a representative example, we show here the results for the incubation with 10 µM of the photocatalyst.

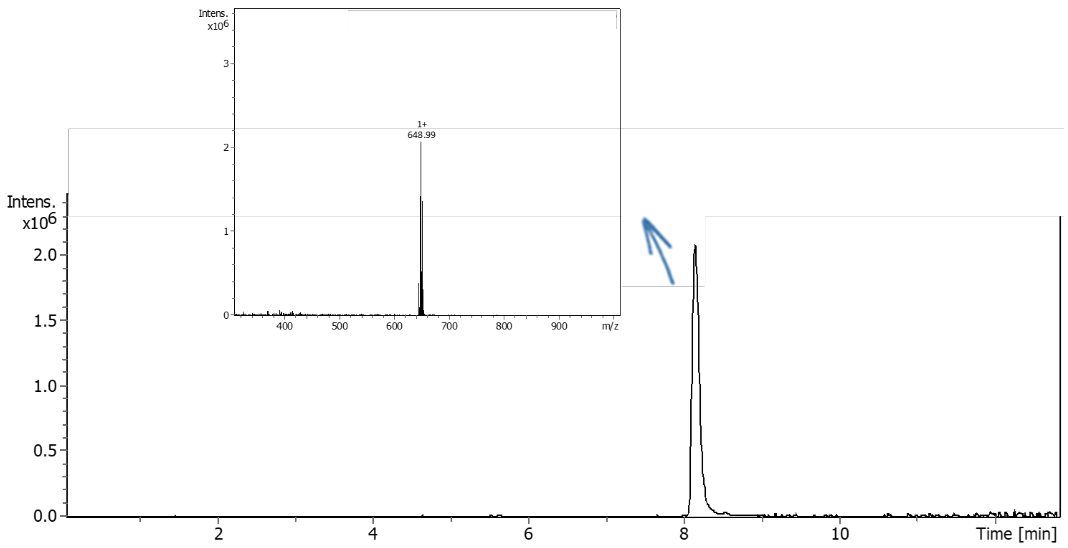

Figure S24. *Representation detection of* ***Eosin Y*** *in HeLa cells (acetonitrile extract), chromatogram with MS detection and inset with the mass spectrum corresponding to the peak. Results when using 10 µM of Eosin Y.*

Representative results using 10 µM of eosin Y

Table S18. Quantification of **Eosin Y** (**10 µM**) inside in **HeLa cells**, after incubation for 15 min. Three experiments performed in different plates with cells proceeding from the same culture.

**Sample**	**Number of cells**	**Value (nmol)**	**Normalization (nmol / 10^6 cells)**
1	$4.4*10^6$	0.120	0.027
2	$4.4*10^6$	0.132	0.030
3	$4.4*10^6$	0.119	0.027
**Average**		0.123 ± 0.008	0.028 ± 0.002

Table S19. Quantification of **Eosin Y** (**10 µM**) inside **HeLa cells** in an experiment performed in one plate.

**Sample**	**Value (nmol)**
Reaction media (DMEM-HEPES)	0.00
Acetonitrile extraction	0.13
**Total value**	0.13

S12. Detection of indole derivative 2b by fluorescence microscopy

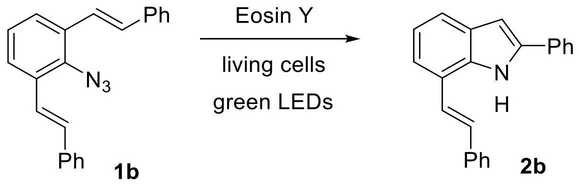

The experiments were performed on glass-bottom plates as follows: 125000 cells per well were seeded on glass-bottom plates two days before treatment. The following conditions reflect the optimized *in cellulo* protocol: Culture medium was replaced by 300 µL of DMEM containing azide **1b** (50 µM) and Eosin Y (5 - 20 µM) or **Eosin-CRGD** (5 - 15 µM). After 15 min of incubation, cells were washed twice with fresh PBS and then treated with HEPES-DMEM (300 µL). Well plates containing cells were disposed as shown in illustration S2, and were irradiated with a Kessil PR160 at 15 cm of distance under fan refrigeration for 15 min.

The cells were observed under microscope with adequate filters. Digital pictures of the samples were taken under identical conditions of gain and exposure.

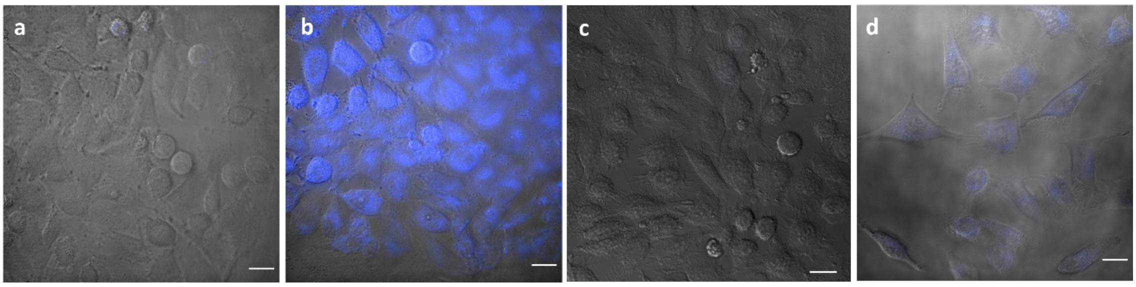

***Figure S25**. Fluorescence micrographies of **control experiments in HeLa cells**, brightfield (a-d) of cells after incubation in **darkness** with **1b** (a), **2b** (b), Eosin Y (25 µM, c) and **1b** and Eosin Y (20 µM, d). Reaction conditions: Cells were pretreated with 50 µM of **1b** or **2b** and Eosin Y (stock solutions in DMEM) for 15 min, washed twice with PBS. Cells were then incubated in HEPES-DMEM for another 15 min. Scale bar: 20 µm. λ_{exc} = 405 nm, λ_{em} = 420-480 nm.*

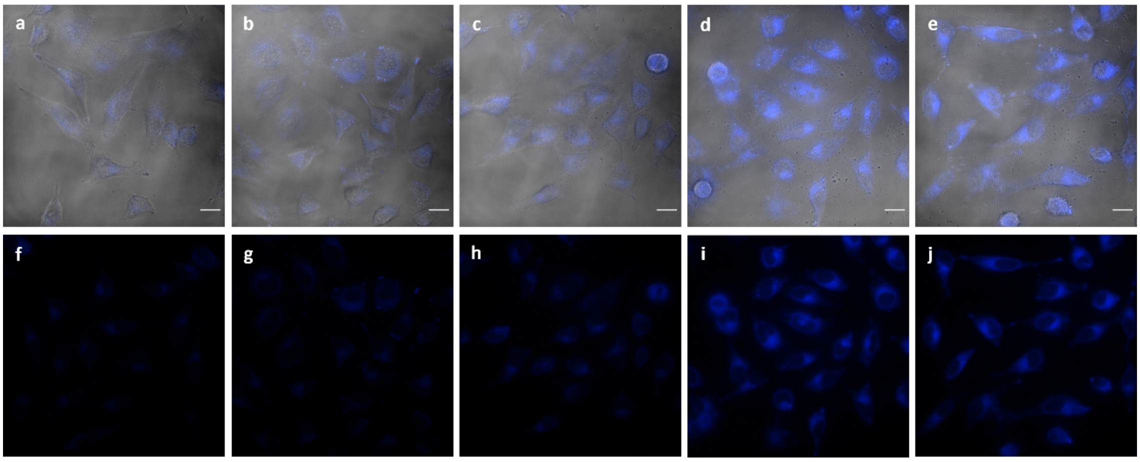

Figure S26. *Fluorescence micrographies of* ***photocatalytic experiments*** *in HeLa cells with increasing amounts of Eosin Y (**from 0 to 20 µM**). Brightfield (a-e) and blue channel (f-j) of cells irradiated with green LEDs after incubation with **1b** (50 µM) and Eosin Y: 0 µM (a,f), 5 µM (b,g), 10 µM (c,h), 15 µM (d,i), 20 µM (e,j). Reaction conditions: Cells were pretreated with 50 µM of **1b** and Eosin Y (stock solutions in DMEM) for 15 min and washed twice with PBS. Cells were then irradiated in HEPES-DMEM under green LEDs for another 15 min. Scale bar: 20 µm.* λ_{exc} = 405 nm, λ_{em} = 420-480 nm.

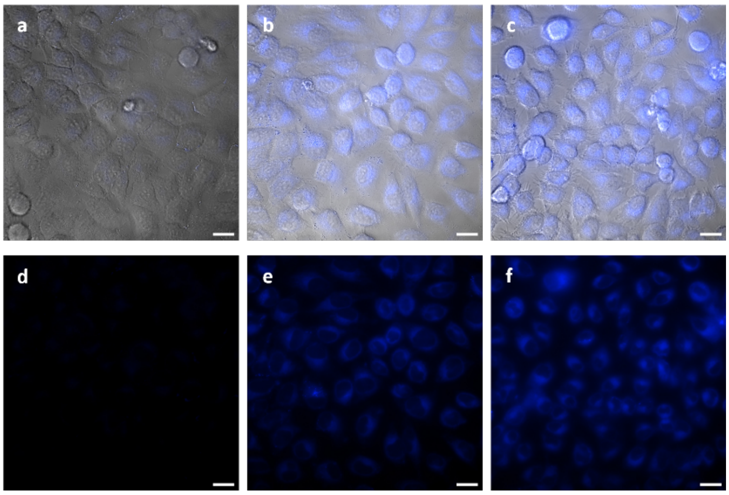

Figure S27. *Fluorescence micrographies of* ***photocatalytic experiments in HeLa cells*** *with increasing amounts of Eosin Y (**from 0 to 50 µM**). Brightfield (a-c) and blue channel (d-f) of cells irradiated with green LEDs after incubation with **1b** (50 µM) and Eosin Y: 0 µM (a,d), 20 µM (b,e), 50 µM (c,f). Reaction conditions: Cells were pretreated with 50 µM of **1b** and Eosin Y (stock solutions in DMEM) for 15 min and washed twice with PBS. Cells were then irradiated in HEPES-DMEM under green LEDs for another 15 min. Scale bar: 20 µm.* λ_{exc} = 405 nm, λ_{em} = 420-480 nm.

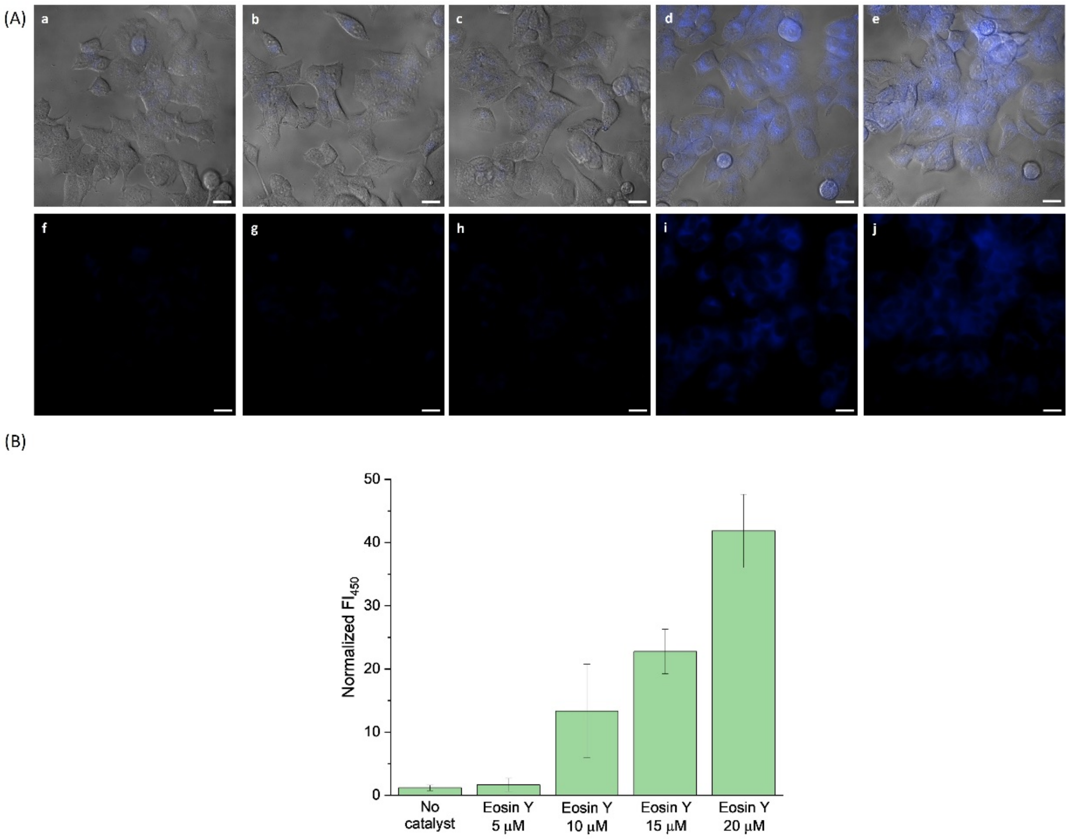

Figure S28. *(A) Fluorescence micrographies of* ***photocatalytic experiments in MCF7 cells*** *with increasing amounts of Eosin Y (from 0 to 20 µM). Brightfield (a-e) and blue channel (f-j) of cells irradiated with green LEDs after incubation with* ***1b*** *(50 µM) and Eosin Y: 0 µM (a,f), 5 µM (b,g), 10 µM (c,h), 15 µM (d,i), 20 µM (e,j). (B) Bar graphic based on CTFC measurements of the intracellular reaction of* ***1b*** *(green bars) with increasing amounts of Eosin Y. Reaction conditions: Cells were pretreated with 50 µM of* ***1b*** *and Eosin Y (stock solutions in DMEM) for 15min and washed twice with PBS. Cells were then irradiated in HEPES-DMEM under green LEDs for another 15 min. Scale bar: 20 µm.* λ_{exc} *= 405 nm,* λ_{em} *= 420-480 nm.*

S13. NMR Spectra

^{1}H NMR ($CDCl_3$, 300 MHz) of (*E*)-2-styrylaniline[1]

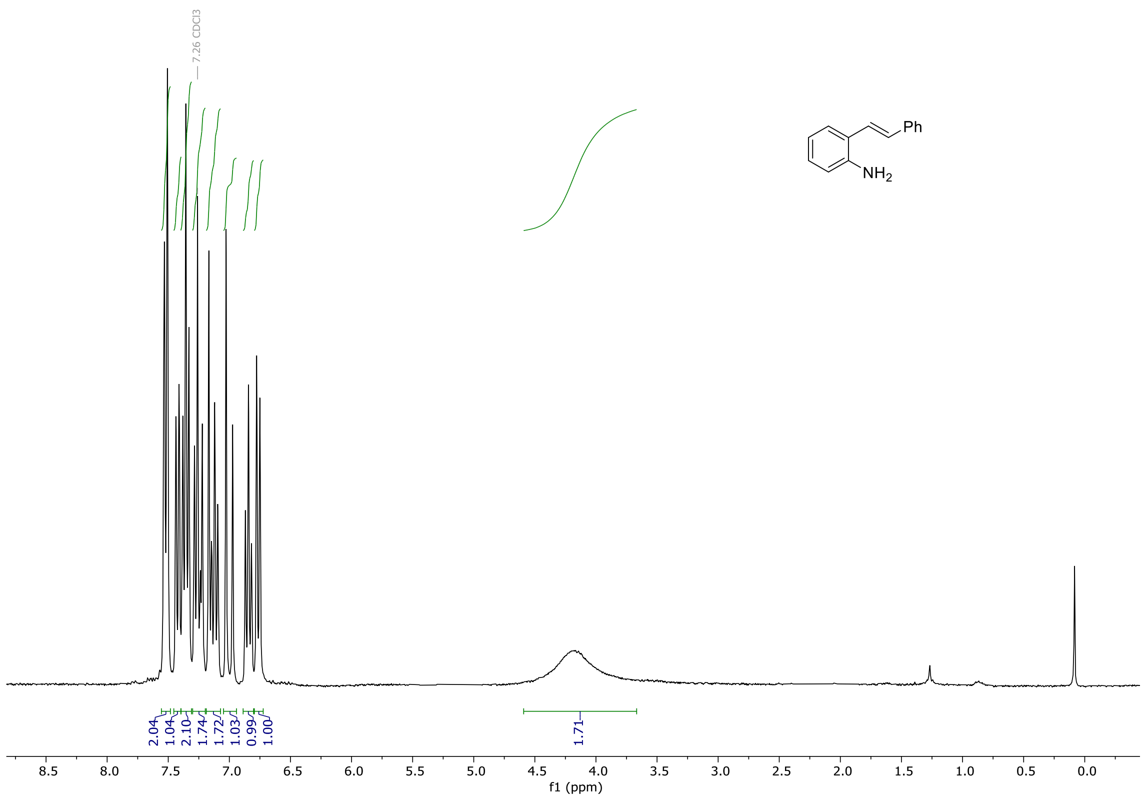

^{1}H NMR ($CDCl_3$, 300 MHz) of (*E*)-1-azido-2-styrylbenzene (1a)[1]

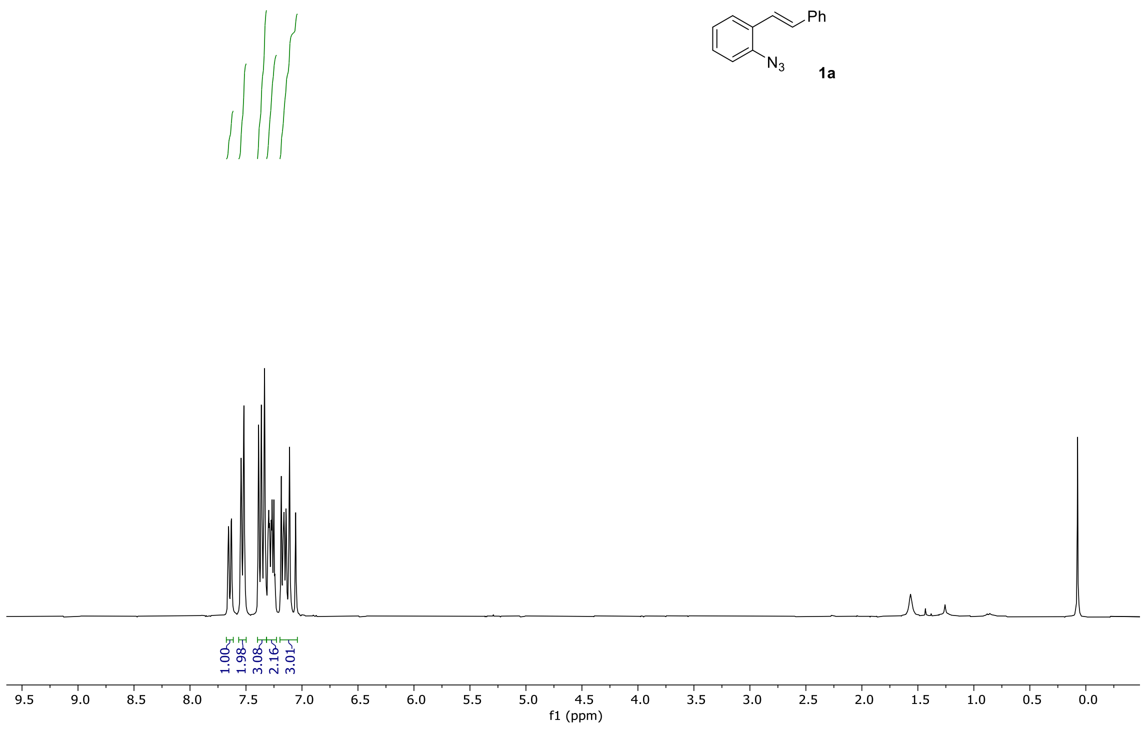

^1H NMR ($CDCl_3$, 300 MHz) of 2-phenyl-1H-indole (2a)[1]

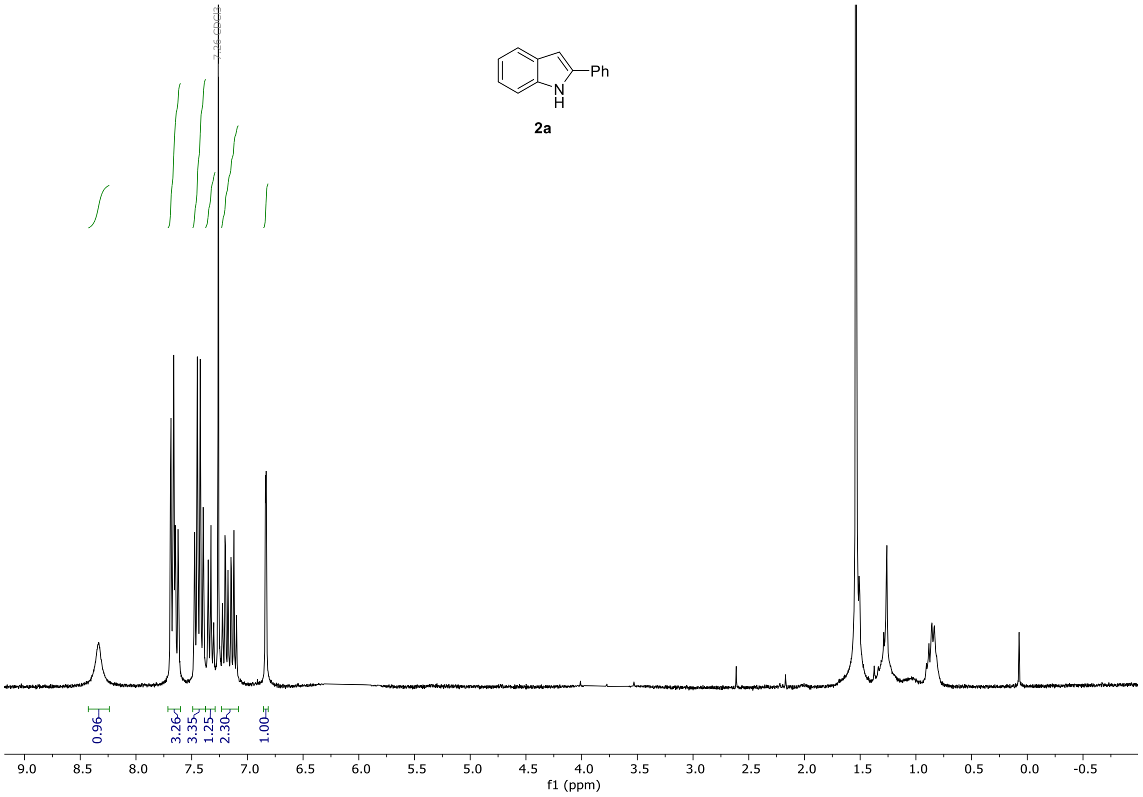

NMR Spectra of 2,6-di[(*E*)-styryl]aniline

^1H NMR ($CDCl_3$, 300 MHz)

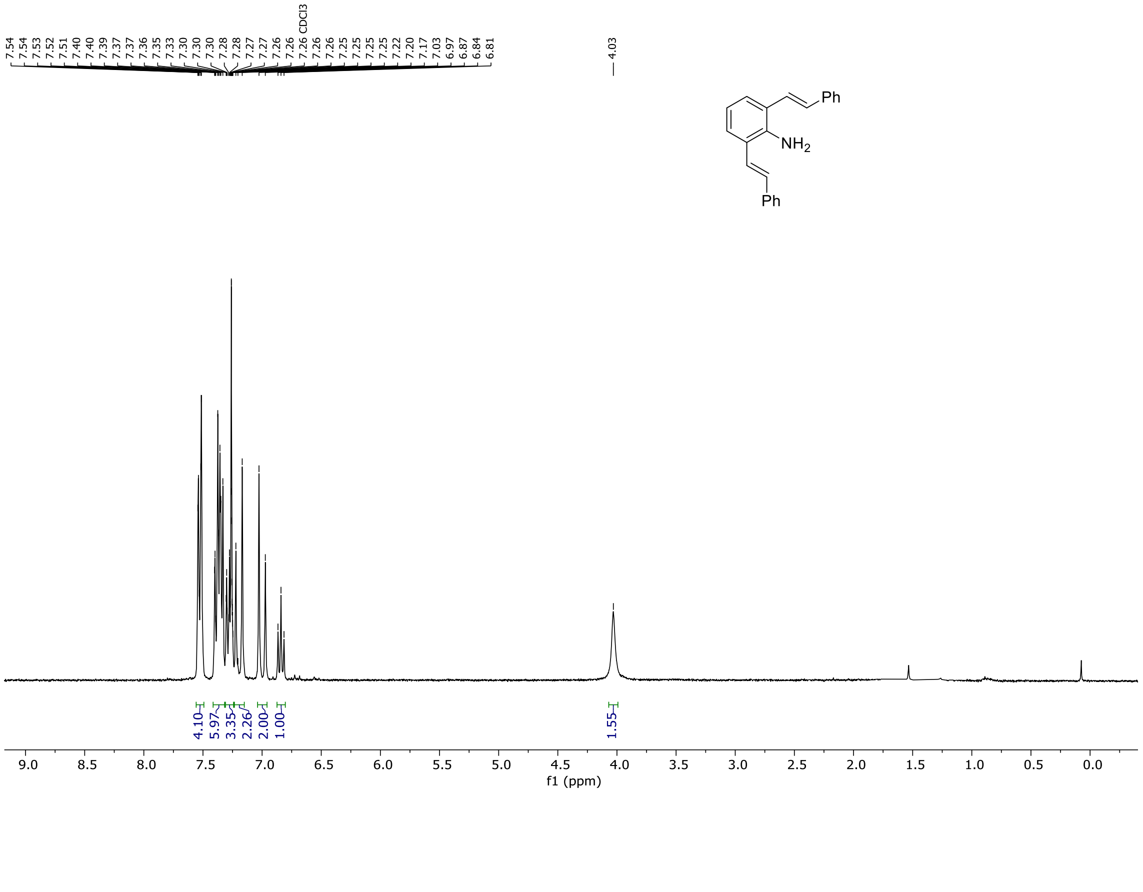

^{13}C NMR ($CDCl_3$, 75 MHz)

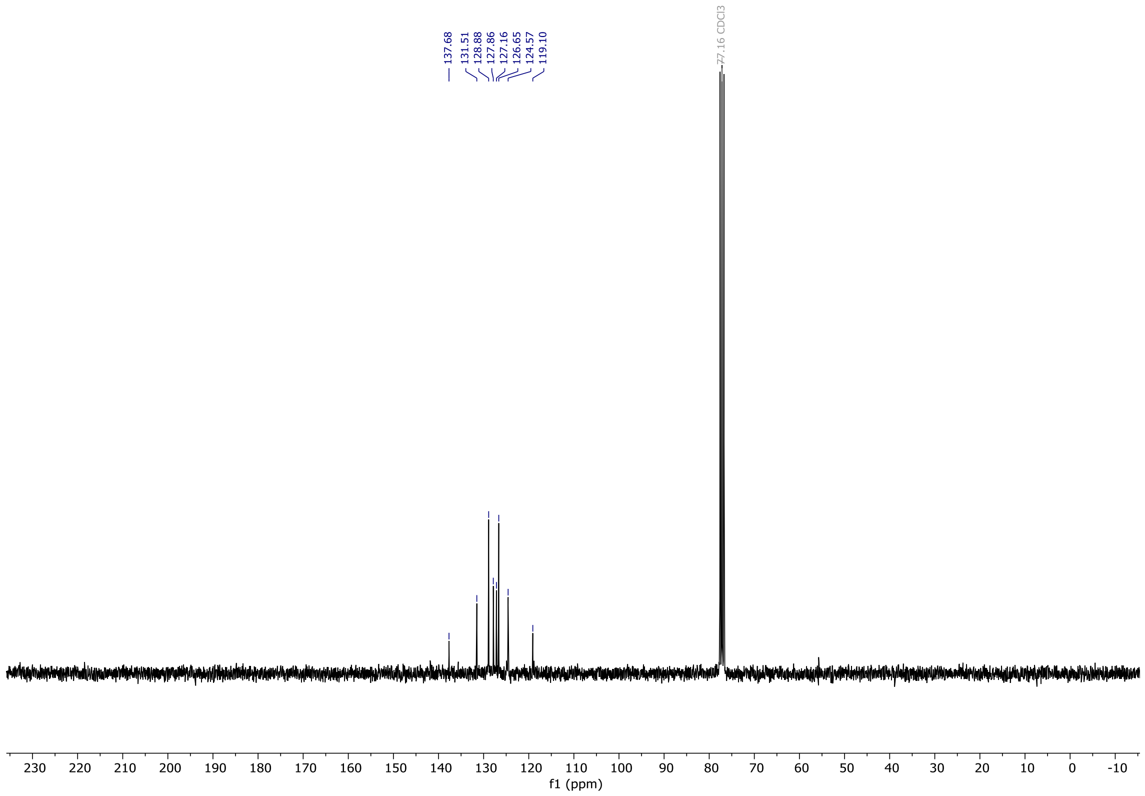

NMR Spectra of [(1*E*,1'*E*)-(2-azido-1,3-phenylene)bis(ethene-2,1-diyl)]dibenzene (1b)

^{1}H NMR ($CDCl_3$, 500 MHz)

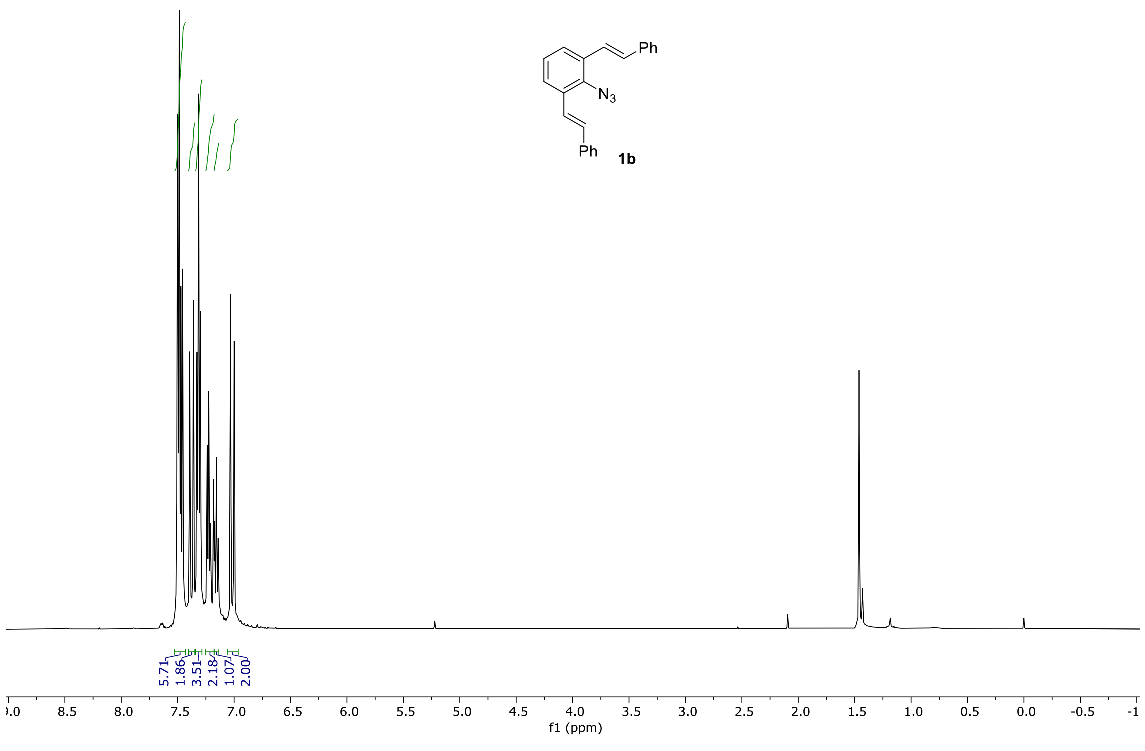

^{13}C NMR ($CDCl_3$, 126 MHz)

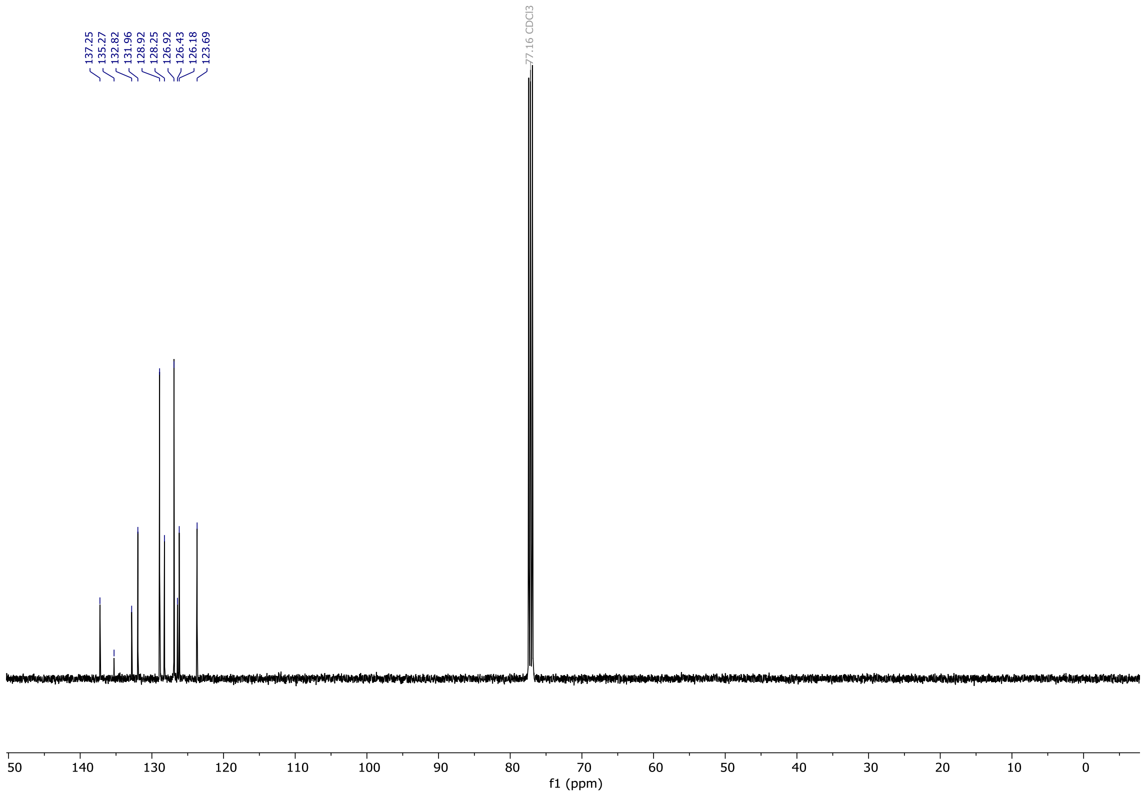

DEPT-135 ($CDCl_3$, 126 MHz)

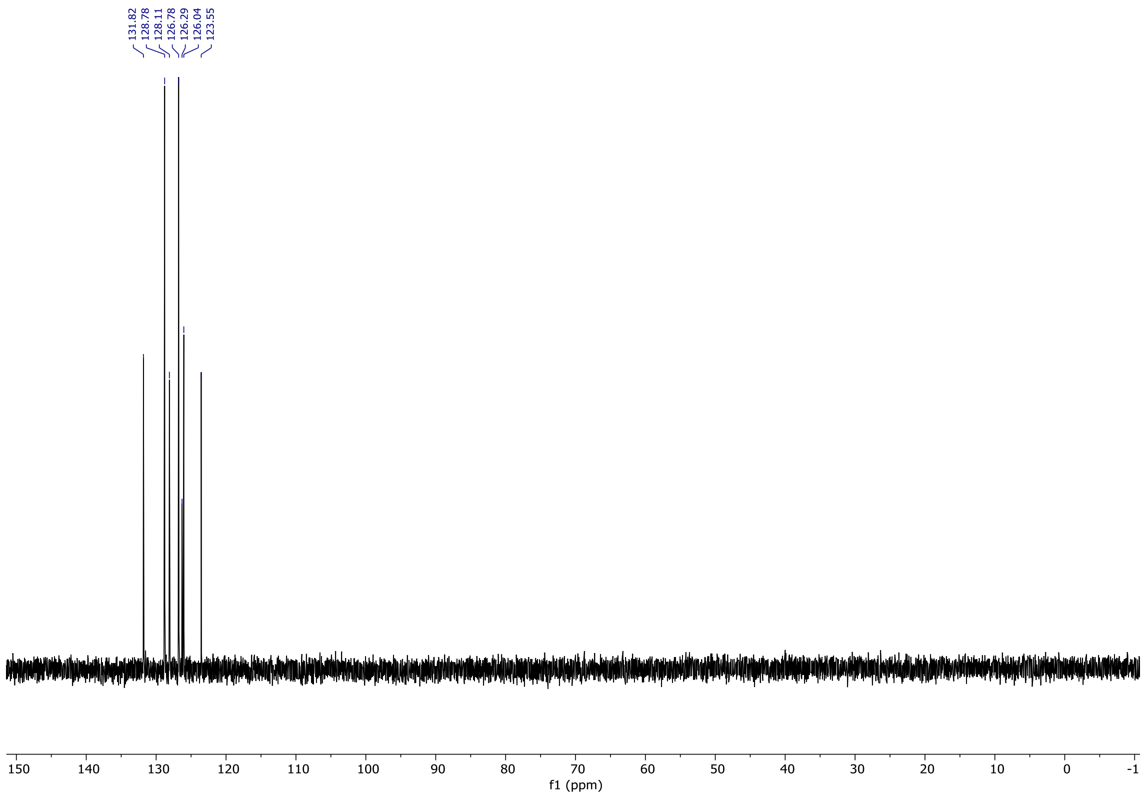

^{1}H NMR (CD_2Cl_2, 500 MHz) of (*E*)-2-phenyl-7-styryl-1H-indole (2b)[3]

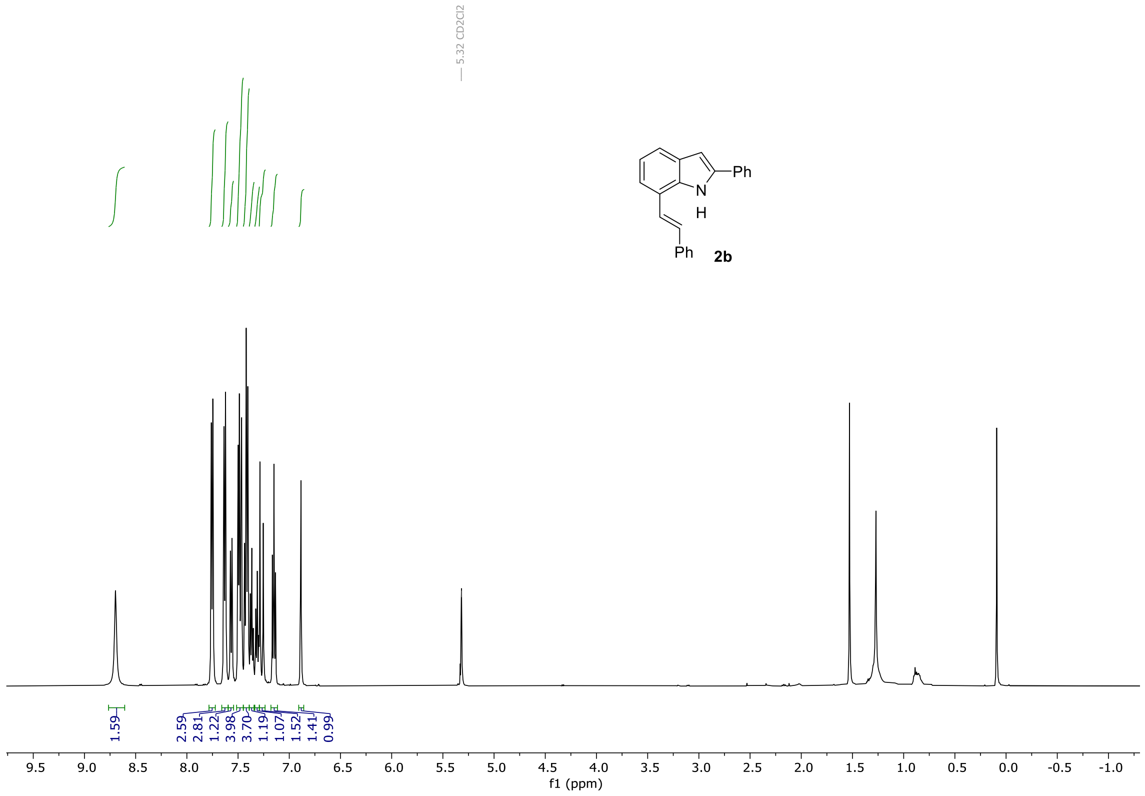

S14. References

[1] Xia, X.; Xuan, J.; Wang, Q.; Lu, L.; Chen, J.; Xiao, W. *Adv. Synth. Cat.* **2014**, *356*, 2807.

[2] Barral, K.; Moorhouse, A. D.; Moses, J. E. *Org. Lett.* **2007**, *9*, 1809-1811.

[3] Zhang, L.; Qiu, R.; Xue, X.; Pan, Y.; Xu, C.; Wang, D.; Wang, X.; Xu, L.; Li, H. *Chem. Commun.* **2014**, *50*, 12385-12388.

[4] Sainlos, M. and Imperiali B. *Nature Protocols* **2007**, *2* (12), 3201-3209.
